# Anti-Diabetic Effects and Mechanisms of Dietary Polysaccharides

**DOI:** 10.3390/molecules24142556

**Published:** 2019-07-13

**Authors:** Kumar Ganesan, Baojun Xu

**Affiliations:** 1 Food Science and Technology Program, Beijing Normal University–Hong Kong Baptist University United International College, Zhuhai 519087, China; 2 Department of Anaesthesiology, Laboratory and Clinical Research Institute for Pain, The University of Hong Kong, Hong Kong, China

**Keywords:** dietary polysaccharides, oxidative stress, molecular mechanisms, anti-diabetic effects

## Abstract

Diabetes mellitus is a multifactorial, heterogeneous metabolic disorder, causing various health complications and economic issues, which apparently impacts the human’s life. Currently, commercial diabetic drugs are clinically managed for diabetic treatment that has definite side effects. Dietary polysaccharides mainly derive from natural sources, including medicinal plants, grains, fruits, vegetables, edible mushroom, and medicinal foods, and possess anti-diabetic potential. Hence, this review summarizes the effects of dietary polysaccharides on diabetes and underlying molecular mechanisms related to inflammatory factors, oxidative stress, and diabetes in various animal models. The analysis of literature and appropriate data on anti-diabetic polysaccharide from electronic databases was conducted. In vivo and in vitro trials have revealed that treatment of these polysaccharides has hypoglycemic, hypolipidemic, antioxidant, and anti-inflammatory effects, which enhance pancreatic *β*-cell mass and alleviates *β*-cell dysfunction. It enhances insulin signaling pathways through insulin receptors and activates the PI3K/Akt pathway, and eventually modulates ERK/JNK/MAPK pathway. In conclusion, dietary polysaccharides can effectively ameliorate hyperglycemia, hyperlipidemia, low-grade inflammation, and oxidative stress in type 2 diabetes mellitus (T2DM), and, thus, consumption of polysaccharides can be a valuable choice for diabetic control.

## 1. Introduction

Diabetes mellitus is a multifactorial metabolic disorder described by chronic hyperglycemia due to insulin resistance or insulin insufficiency [1]. It is a heterogeneous disorder that can potentially cause insulin resistance in the peripheral tissues, including adipose, liver, and muscle, as well as progressive *β*-cell dysfunction in the islets of the pancreas, resulting in hyperglycemia [2]. In a patient with type 2 diabetes mellitus (T2DM), it is estimated that almost 90% of all the patients with diabetes show hyperglycemia, impaired glucose tolerance, dyslipidemia, hyperinsulinemia, and persistent insulin deficiency [3]. Moreover, the long-lasting complications of T2DM cause extraordinary healthcare costs with high morbidity and mortality [2,4]. Administration of oral hypoglycemic agents, such as biguanides, thiazolidinediones, and sulfonylureas, can meritoriously regulate hyperglycemia. Nevertheless, they have noticeable side-effects, including hypoglycemia and gastrointestinal troubles [5]. Thus, there is an urgent requirement for effective substitutions to reduce the complications of diabetes with lower side-effects. In recent years, the search for alternate medications has drawn great attention to combat diabetes. Therefore, dietary active principles progressively come into the field of investigator’s vision [4]. Dietary active principles, such as polysaccharides, alkaloids, steroids, polyphenol, terpenoids, glycoproteins, and saponins, have medicinal value and have been connected with various pharmacological effects, such as improving immune function, anti-diabetic, antioxidant, anti-cancer, anti-aging, antimicrobial, and anti-inflammatory properties [6,7,8,9].

**Figure 1:**
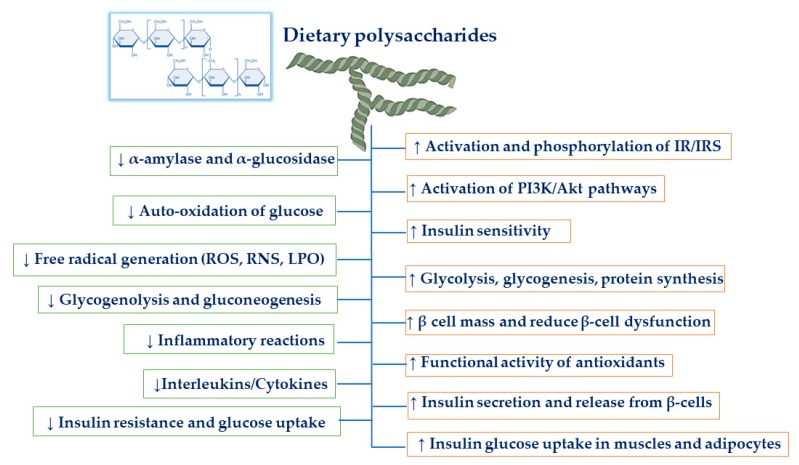
Dietary polysaccharides exert favorable effects on glucose homeostasis and reduce insulin resistance. IR: insulin receptor, IRS: insulin receptor substrate, PI3K: phosphoinositide 3-kinase, AKT: serine/threonine-specific protein kinase, ROS: reactive oxygen species, RNS: reactive nitrogen species, LPO: lipid peroxidation.

Dietary polysaccharides are natural edible polysaccharides that are essential for our day to day life. Polysaccharides generally extracted from medicinal plants, grains, fruits, vegetables, edible mushroom, and medicinal foods have shown more consideration from investigators due to their low toxicity and numerous pharmacological activities [10,11,12]. They are composed of the monosaccharide unit and linked by glycosidic bonds. The assessment of the effects of polysaccharides with anti-diabetic properties has emerged as an important research field [10]. Various investigations have presented that polysaccharides purified from pumpkin, sea cucumber, goji berry, mushroom, bean, tea, and oat exert favorable effects on glucose homeostasis (Figure 1), reduce the complications of diabetes through the defensive mechanism against oxidative stress injury, and eventually improve insulin sensitivity [13,14,15]. In addition, dietary non-digestible polysaccharides obtained from plants and foodstuff are recognized potent modulators of gut microbiota that can nourish certain useful microbes in the human gut. This has also led to increased attention to the isolation of novel bioactive polysaccharides and their practices as functional components, which modulate the gut microbiota to improve the host’s metabolism and health [1]. Hence, polysaccharides are considered prebiotic substances, which are being speculated to confer positive effects in managing metabolic diseases like diabetes. In this comprehensive review, we summarize some of the most common dietary polysaccharides from medicinal plants, grains, fruits, vegetables, edible mushroom, and medicinal foods that impact metabolic health and discuss underlying molecular mechanisms, related to oxidative stress and inflammatory factors, which could be supportive in ameliorating type-2 diabetes.

## 2. Anti-Diabetic Potentials of Polysaccharides

Dietary polysaccharides are largely obtained from natural sources, including medicinal plants, grains, fruits, vegetables, mushroom, medicinal foods, algae, and fungi. They have potential anti-diabetic effects with underlying various molecular mechanisms to combat diabetic complications. Polysaccharides proceed to regulate hyperglycemia primarily established on their sources, composition, and preparation. Polysaccharides have been documented to have potent anti-diabetic activity. *β*-d-(1→6)-glucan can improve the insulin level and hepatic glycogen accumulation, decreasing the blood glucose level in streptozotocin (STZ)-induced diabetic mice [14]. A polysaccharide purified from *Lycium barbarum* has a composition of mainly mannose, rhamnose, and glucose. It can inhibit the absorption of glucose in a dose-dependent manner [16]. *β*-d-glucan of *Agaricus blazei* (MW: 136.05 kDa), *Trametes gibbosa* (MW: 3.872, 2.761, 8.526, and 5.659 kDa), *Saccharina japonica* (MW: 7.28 kDa), and *Lachnum calyculiforme* (MW: 445.363 kDa) has been documented to have hypoglycemic, hypotriglyceridemic, and hypocholesterolemic activities in diabetic rats. Moreover, the oligosaccharides hydrolyzed from *β*-d-glucan show diabetic improved activities [17,18,19,20]. A crude extract of *Talinum triangulare* polysaccharide contains rhamnose, arabinose, mannose, and galactose, which demonstrated an anti-diabetic effect in STZ diabetic rats [21]. Similarly, aqueous-soluble polysaccharides (MW: 60, 350, and 3000 kDa) obtained from *Schisandra chinensis* (Turcz.) Baill (ESCPs) is composed of L-rhamnose, L-arabinose, D-xylose, D-glucose, D-galactose, and D-mannose that evidently decreased the blood glucose level in alloxan-induced diabetic mice after 21-day oral administration [13]. A sulfated polysaccharide purified from *Saccharina japonica* primarily comprises fucose, sulfate uronic acid, galactose, mannose, glucose, and arabinose, which exhibited a potential hypoglycemic effect by markedly decreasing blood glucose and augmenting insulin levels in alloxan-induced diabetic mice [19]. Two polysaccharides (MW: 0.7, 3.5 kDa) obtained from *Inula britannica* (MW: 0.7, 3.5 kDa) contains mannose, glucuronic acid, rhamnose, galacturonic acid, glucose, galactose, and arabinose that markedly decreased the plasma glucose level and increased the liver glycogen content in alloxan-induced diabetic mice [22]. The anti-diabetic effects of various grains, fruits, vegetables, edible mushrooms, and medicinal foods have been listed in Table 1.

## 3. Mechanism of Dietary Polysaccharides on Anti-Diabetic Activities

Polysaccharides are generally extracted from dietary materials by various physical, chemical, or enzymatic digestion treatments that can be found to have anti-diabetic potentials. The previous study showed that polysaccharides consumption could alleviate diabetes through mechanisms of action on gastrointestinal viscosity, gastrointestinal satiety, colon fermentation, and anti-gastrointestinal inflammation [68]. Similarly, the present study aimed to identify the various in vivo and in vitro trials in which dietary polysaccharides have hypoglycemic, antioxidant, and anti-inflammatory effects. Dietary polysaccharides enhance pancreatic *β*-cell mass, trigger insulin signaling pathways through insulin receptors, and activate the PI3K/Akt pathway. They modulate ERK/JNK/MAPK pathways and, thus, alleviate *β*-cell dysfunction.

### 3.1. Hypoglycemic and Hypolipidemic Effects

Impaired glucose tolerance generally leads to permanent loss of *β*-cell function, which has been recognized by the occurrence of glucose toxicity and lipotoxicity. Hyperglycemia often produces an elevated reactive oxygen species in *β*-cells, providing succeeding impairment to cellular mechanisms [69,70]. The pancreatic lipid contents generally not relate with *β*-cell dysfunction in young-onset T2DM [71] and, however, pancreatic islet lipotoxicity is known as a major factor for the onset and progression of T2DM. The disorder of lipid metabolism or increasing fatty acid levels in the blood cause *β*-cell dysfunction, which are primary threat factors for T2DM [39]. An investigation of animal study connected with streptozotocin-induced diabetes succeeding *β*-cell dysfunction suggested that the polysaccharide obtained from gum exudates (*Acacia tortilis*), comprising of the polymer compounds (D-glucose, D-galactose, L-rhamnose, and D-glucuronic acid), showed a potential anti-diabetic effect. These extracts remarkably decreased fasting blood glucose (FBG), glycated hemoglobin (HbA1c), total cholesterol (TC), triglyceride (TG), low-density lipoprotein (LDL), very LDL (VLDL), and an elevated concentration of high-density lipoprotein (HDL). Furthermore, reduced hepatic markers, such as aspartate transaminase (AST) and alanine transaminase (ALT), were noticed in gum extract treated groups, indicating improved lipid metabolism in the liver [72]. Various animal studies reported that well characterized, low to high molecular weight polysaccharides obtained from various edible sources showed greater anti-diabetic effects [13,18,73]. A polysaccharide purified and characterized by *Lachnum calyculiforme* demonstrated a significant hypoglycemic effect (*p* < 0.01) at dose-dependent manners in alloxan-induced diabetic mice [18]. Similarly, corn silk, pumpkin, *Mactra veneriformis*, *Trametes gibbosa, Inula britannica, Saccharina japonica, Phellinus linteus, Talinum triangulare,* and *Schisandra chinensis* (Turcz.) Baill containing different ranges of molecular weight polysaccharides (60, 180, 220, 350, 449.6, 3000, 3172.9 kDa) exhibited a significant anti-diabetic effect (*p* < 0.05) as proved by a remarkable decrease of blood glucose and improvement of OGTT, serum insulin, and lipid metabolism outcomes in STZ as well as alloxan-induced diabetic mice [13,22,73,74,75].

The fruit polysaccharide of *Morus alba* L. has significant anti-hyperglycemic and anti-hyperlipidemic effects that can undoubtedly relieve symptoms of diabetes in the STZ-induced T2DM rat model. After seven weeks’ treatment, the fruit polysaccharides significantly diminished FBG, OGTT, glycated serum protein, and lipid profiles and improved insulin levels in the blood. Furthermore, the polysaccharides-treated groups enhanced the insulin-signaling pathway, and their high protein expression levels of InsR (insulin receptor), IRS-2 (insulin receptor substrate 2,), Akt (serine/threonine-specific protein kinase), and GLUT4 (glucose transporter 4) were identified when compared to that of the T2DM groups [76]. High-fat diet treated with STZ-induced hyperglycemia was significantly reduced by the administration of mushroom polysaccharides [77,78]. The oral administration of mushroom extracellular polysaccharides obtained from *Pleurotus tuber*-*regium* (20 mg/kg b.w. (body weight)) and *Grifola frondosa* (100 or 300 mg/kg b.w.) could decline the levels of FBG, TC, TG, lipid profiles, fatty acid composition, and expression of liver peroxisome proliferator-activated receptor alpha (PPAR-α) in obese-diabetic rats. The parallel restoration and elevated HDL-C levels occurred with supplementation of mushroom polysaccharides [77,78]. These hypolipidemic properties might be connected with up-regulated expression of liver PPAR-α mRNA and protein levels [78]. All these outcomes strongly suggest that polysaccharides exert potential hypoglycemic and hypolipidemic effects in STZ-induced diabetic animal models. Therefore, polysaccharides could be considered as a nutritional supplement to treat diabetic complications.

### 3.2. Increasing β-Cell Mass and Reducing β-Cell Dysfunction

A recent study described that lean and obese human, with T2DM, had a 45% and 70% decreased relative *β*-cell mass; 10- and 3-fold elevated *β*-cell apoptosis, respectively, compared with the respective nondiabetic control group [79,80,81]. This research outcome suggested that the decreased *β*-cell mass along with elevated *β*-cell apoptosis rate is relatively common in T2DM. Though the underlying mechanism of *β*-cell apoptosis in T2DM is complex and debated [82,83], the prevention of *β*-cell apoptosis and connected elements are the vital approach for treating T2DM.

Several animal studies reported that purified, characterized low to high molecular weight polysaccharides obtained from dietary sources showed elevating *β*-cell mass and reducing *β*-cell dysfunction [84,85]. The oral administration of mulberry leaf containing polysaccharides (MW: 8.1 kDa) significantly prevented *β*-cell apoptosis and elevated insulin secretion in STZ-induced diabetic rats. These polysaccharides significantly up-regulated Bcl-2 (B-cell lymphoma 2) and PDX-1 (insulin promoter factor 1) and down-regulated mRNA expression of Bax (BCL2 associated X protein). In addition, they markedly prevented caspase-3 activation in the islets of the pancreas of STZ- diabetic rats [84]. These results suggested that polysaccharides could play a critical function in pancreatic islet cell protection from apoptosis by increasing the ratio of Bcl-2/Bax and improving insulin secretion through the restoration of PDX-1 in diabetic animals [84]. Similarly, a polysaccharide from *Ganoderma atrum* (MW: 1013 kDa) administration in diabetic animals significantly reduced FBG, plasma insulin, and expression of Bax and improved expression of Bcl2 as well as lipid profiles in the high-fat diet STZ-induced diabetic rats [85]. Histopathological studies also confirmed that polysaccharides from *G. atrum* showed elevated *β*-cell mass, pancreatic islets expansion, and restoration, representing that polysaccharides protected the islets of the pancreas from HFD- and STZ-induced damage [86]. Another study from fruit bodies of *Ganoderma lucidum* containing protein-bound polysaccharide (MW: 8.849 kDa) exhibited potential anti-hyperglycemic and anti-hyperlipidemic effects on STZ-induced diabetic rats [87]. The underlying mechanism of this study observed that *G. lucidum* significantly up-regulated Bcl-2 and down-regulated Bax and caspase in the pancreatic cells compared to that of STZ diabetic animals. The results strongly suggested that polysaccharide from *G. lucidum* exerted an anti-diabetic potential by inhibiting the *β*-cell apoptosis in diabetic rats [88].

Zhu et al. [89] demonstrated a low molecular weight and well-characterized polysaccharide from pumpkin fruit (MW: 115 kDa) that prevented *β*-cell apoptosis by regulating the mRNA expression of Bcl-2 and Bax in STZ-induced damage of pancreatic islet cells. They found that polysaccharides from pumpkin possessed strong antioxidant capacities and eventually decreased the NO level and restore the *β*-cells. Zhang et al. [34] also presented that water-soluble polysaccharide purified from pumpkin restored the damaged pancreatic islets via triggering *β*-cell multiplication. This investigation further observed that intragastric treatment of polysaccharide from pumpkin significantly decreased blood glucose, TC, TG, and HbA1c in alloxan-induced diabetic animals and restored the normalization within 21 days’ treatment of polysaccharides. A low molecular weight polysaccharide purified from Sea cucumber (*Cucumaria frondosa*, MW: 14.76 kDa) and *Lycium barbarum* L (goji berry, MW: 212.14 kDa) prevented *β*-cell apoptosis and increased *β*-cell mass in pancreatic islets of mice by down-regulating the mitochondrial signaling pathway, eventually showing significant insulin-sensitizing and anti-hyperglycemic effects [86,90]. All these findings recommend that polysaccharides aid in increasing *β*-cell mass and reducing *β*-cell dysfunction. Hence, polysaccharides could be considered as a dietary supplement involved in the pathogenesis of diabetes, leading to reduce the degree of *β*-cell damage in pancreatic islets.

### 3.3. Antioxidant Effects

Diabetes is generally caused by the impairment or insufficient *β*-cells in the pancreas that diminishes insulin biosynthesis and gradually deteriorates whole body functions. In contrast to physiological glucose concentration, these glucose levels negatively affect a greater number of organs and tissues. Due to chronic hyperglycemia, decreasing insulin secretion, as well as up-surging insulin resistance, provides glucose toxicity [81,91,92]. It has been recognized that glucose toxicity mainly participates in the deterioration of diabetes by distressing the synthesis of *β*-cells. The underlying mechanism of glucotoxicity is chiefly mediated by oxidative stress, which has been associated as a primary risk factor in the onset and advancement of T2DM. Oxidative stress is usually formed by an excess free radical formation and decreases the antioxidant defense system in the body [81]. Generally, the living system generates two major forms of reactive species, viz. reactive oxygen species and reactive nitrogen species. Pancreatic *β*-cells are susceptible to those reactive species due to their low concentration of free-radical scavenging enzymes. They can readily injure to cellular macromolecules, such as lipids, DNA, and proteins [93]. However, the antioxidant agents derived from dietary sources are promising elements to scavenge cell generating free radicals and protects *β*-cells. These antioxidant agents generally augment cellular antioxidant enzymes and inhibit *β*-cell apoptosis, which has been demonstrated to improve *β*-cell dysfunction and protect *β*-cells against glucotoxicity in diabetic animal models [5,94,95].

In vivo, animal studies demonstrated that bioactive polysaccharide could inhibit the development of T2DM by decreasing oxidative stress. For instance, the polysaccharide obtained from *Grifola frondosa* (MW: 400–450 kDa) and *Salvia miltiorrhiza* Bunge (MW: 119.5 kDa) showed substantial defensive and antioxidative ability against the oxidative damage and increased the activities of antioxidant enzymes, such as SOD (superoxide dismutase), CAT (catalase), and GSH-Px (glutathione peroxidase) and decreased level of malondialdehyde (MDA), NO synthase, and inducible NOS (Nitric oxide synthase) in blood and liver [96,97]. MDA is measured as a fundamental chain reaction of lipid peroxidation, which produces injury to the cell membrane, necrosis, and inflammation [92]. Additionally, *G. frondosa* and *S. miltiorrhiza* improved the insulin sensitivity index and attenuated STZ-induced structural changes to the pancreas and liver [96,97]. Similarly, the low molecular weight polysaccharides from *Catathelasma ventricosum* (MW: 160 kDa) and *Ophiopogon japonica* demonstrated anti-diabetic, anti-obesity, and antioxidant activities in STZ-induced diabetic mice. Oral administration of both plants decreased the MDA levels and increased vitamin E contents, SOD, CAT, and GSH-Px activities in the hepatic and renal cells of STZ-induced diabetic mice [14,98]. Simultaneously, oral administration of these polysaccharides significantly decreased blood glucose and markedly elevated serum insulin levels. Microscopic observation in the pancreas, kidneys, and liver assay confirmed that polysaccharides protected the organs from lipid peroxidation injury and conserved tissue integrity [99]. The enhancement of antioxidant enzyme activity in the treated group designated that polysaccharides inhibited the cell injury by scavenging free radicals produced by chain reactions of lipid peroxidation [70].

Mulberry fruit polysaccharide, produced by Fructus Mori, is a biopolymer that exhibited hypoglycemic and antioxidant activities in vitro as well as in STZ-induced diabetic mice. In vitro, hypoglycemic experiments exhibited that a noteworthy insulin-sensitizing and increased insulin synthesis was observed when the treatment with polysaccharides stimulated pancreatic *β* cell proliferation and serum insulin levels. Oral administration of fruit polysaccharide could markedly decrease blood glucose and MDA levels and increase SOD, CAT, and GSH-Px in the hepatocytes of STZ-induced diabetic mice. Histopathological observation exhibited that fruit polysaccharide could significantly improve the tissue damage to the pancreas, liver, and kidney [100]. Likewise, ginseng polysaccharides from *Panax ginseng* C.A. Meyer showed significant hypoglycemic and antioxidant activities in STZ-induced diabetic mice. Oral administration of ginseng polysaccharides significantly decreased blood glucose and lipid peroxidation levels and enhanced SOD levels [101].

Another study in white oyster culinary-medicinal mushroom polysaccharide, which was obtained from *Pleurotus florida* (MW: 155 kDa), showed decreased blood glucose, HbA1c, lipid profiles, and urinary glucose in STZ- induced diabetic rats. *P. florida* decreased the levels of MDA and nitric oxide and restored the levels of GSH, SOD, and CAT in diabetic rats. These findings recommended that administration of *P. florida* could attenuate diabetic complications along with hyperglycemia and hypercholesteremic effects [102]. Based on the investigations, all these results strongly suggest that polysaccharides possess antioxidant properties that can be applied as an adjunct therapy and control the effect of T2DM.

### 3.4. Anti-Cholesterolemic and Anti-Triglyceridemic Effects

Various in vitro and in vivo studies demonstrated that dietary polysaccharides potentially have lipid-lowering effects and eventually reduce the effects of diabetic complications. Polysaccharides activate serine/threonine protein kinase (AMPK) pathway to regulate lipid metabolism by decreasing the levels of triglycerides and cholesterol. Studies have revealed that AMPK switches off anabolic processes, including the biosynthesis of fatty acids, triglyceride, and cholesterol, through repressing the expression of genes, such as Acetyl-CoA carboxylase (ACC), sterol regulatory element binding protein -1c (SREBP-1c), and 3-hydroxy-3-methylglutaryl-CoA (HMG-CoA) reductase [103,104,105].

Normally, the reduction of triglycerides occurs in the human body through activation of an enzyme, adipose triglyceride lipase, or up-regulation of peroxisome proliferator-activated receptor-α (PPAR-α) and PPAR gamma coactivator-1 alpha pathways [106,107]. These signaling pathways are highly connected with energy expenditure as well as reduce the uptake of energy substrates. Furthermore, triglyceride levels can be decreased by triggering another enzyme, ACC, and up-regulation of SREBP-1c or down-regulation of FAS (fatty acid synthase)-carnitine palmitoyltransferase-1 (CPT1) signaling pathways. Studies have shown that over-expression of CPT1 can elevate fatty acid oxidation, lessen cellular triglyceride accumulation, and reduce high-fat-diet-induced insulin resistance [107,108]. The activity of CPT1 is generally regulated by ACC, through the manufacturing of malonyl-CoA, which acts as an inhibitor of CPT1 [108].

The consumption of dietary polysaccharides can be another effective treatment or can prevent hypercholesterolemia. The dietary polysaccharides exert cholesterol lowering effects via activation of sterol regulatory element binding protein -2 (SREBP-2) or inhibition of rate-limiting enzyme, 3-hydroxy-3-methylglutaryl-CoA reductase (HMG-CoA reductase). The expression of HMG-CoA reductase gene is controlled by the SREBP, which play vital roles in managing the biosynthesis of cholesterol and fatty acids. Dietary polysaccharides have inhibitory effects on HMG-CoA reductase through the activation of SREBP-2 [107,109,110,111]. Increasing plasma LDL-cholesterol (LDL-C) level is a major cardiovascular risk for diabetic patients [112,113]. Uptake of these LDL-C through LDL receptor-mediated endocytosis is an essential step for the regulation of cholesterol homeostasis [114]. Hence, the activation of the LDL receptor reduces the plasma LDL-C levels. Proprotein convertase subtilisin/kexin type 9 is a class of proteinase, that can usually degrade LDL receptor resulting in the elevation of LDL-C in the blood [115]. Dietary polysaccharides are known to activate LDL receptors via inhibiting this enzyme and prevent the elevated LDL-C levels in diabetic patients [111]. Anti-cholesterolemic and anti-triglyceridemic effects of dietary polysaccharides are listed in Table 2. Based on the studies, all these outcomes strongly suggest that polysaccharides possess anti-cholesterolemic and anti-triglyceridemic effects that can be applied as an adjunct therapy for CVD (Cardiovascular diseases) and control the effect of T2DM.

### 3.5. Anti-Inflammatory Effects

In general, oxidative stress is connected with chronic inflammation in T2DM. During inflammation, several pro-inflammatory cytokines, namely, interleukin (IL)-1, IL-6, IL-8, IL-12, IL-18, interferon gamma (IF-γ), and tumor necrosis factor alpha (TNF-α), serve key functions in the dysfunction of islet cells and insulin receptors in the pancreas and eventually *β*-cell death [3,4,95,121]. Studies have shown that treatment using anti-inflammatory agents, including IL-1 and receptor antagonists, normalize glucose in the blood, improve insulin secretion, and decrease inflammation causing islet fibrosis. These findings suggested the improvement of *β*-cell dysfunction and cell survival [122].

Various preclinical studies validated that bioactive polysaccharide can inhibit the progression of T2DM by decreasing pro-inflammatory factors. A high molecular weight polysaccharide (MW: 72.9 kDa), which was obtained from the roots of *Angelica sinensis* (Oliv.) Diels, exhibited hypoglycemic and hypolipidemic effects in STZ-induced diabetic mice. These root polysaccharides markedly exhibited anti-inflammatory effects by decreasing the insulin receptor-associated inflammatory factors, including IL-6 and TNF-α, in STZ-induced diabetic mice [123]. Generally, IL-6 is synthesized by macrophages and T cells for triggering immune responses to the host. IL-6 can also certainly avert insulin synthesis, and excess IL-6 leads to severe pancreatic islet cytotoxicity and cause insulin resistance [4]. TNF-α is a well-recognized cell signaling protein, which is certainly correlated with the insulin receptor and *β*-cell dysfunction and vastly articulated in adipose cells [124,125]. Animal studies connected with T2DM exhibited that the polysaccharide from *Rehmannia glutinosa* (Gaertn.) DC (MW: 63.5 kDa) meritoriously improved hyperglycemia, vascular inflammation, hyperlipidemia, and oxidative stress [124].

A low molecular weight polysaccharide (MW: 50–210 kDa) acquired from *Pseudostellaria heterophylla* demonstrated a hypoglycemic potential in STZ-induced type 2 diabetic rats. These polysaccharides improved insulin sensitivity and markedly decreased lipid profiles and TNF-α expression and elevated IL-10 concentration. IL-10 has generally pleiotropic properties on inflammation, in which the polysaccharides avert the inflammatory mechanism by condensing the secretion and activities of proinflammatory cytokines. These findings clearly exhibited that polysaccharides attenuated low-grade inflammation connected with T2DM [126]. Similarly, the fruit body of mushroom polysaccharide from *Pleurotus sajor-caju* and *Ramulus mori* and the polysaccharide extracted from *Morus alba* L. reduced blood glucose and attenuated hyperglycemia and hyperinsulinemia in diabetic mice. In addition, the polysaccharides decreased the expression of various proinflammatory cytokines, including IL-6, IL-8, COX-2 (cyclooxygenase-2), TNF-α, by down-regulating the signaling of nuclear factor kappa B (NF-kB) [127,128]. Preventing these inflammatory factors are normally positive strategies for averting or alleviating pancreatic islet damage and reducing T2DM development. Hence, polysaccharides greatly normalize the pancreatic function from STZ-induced damage, and this normalization could be connected with a reduction of inflammatory factors and oxidative stress in pancreatic islets [129].

Various low molecular weight polysaccharides extracted and purified from *Misgurnus anguillicaudatus* (MW: 130 kDa), *Anoectochilus roxburghii, Vigna radiata* L., and *Hedysarum polybotrys* demonstrated anti-hyperglycemic, antioxidant, anti-inflammatory, and anti-hyperlipidemic effects in diabetic animals, which was reflected by decreased blood glucose, MDA, MCP-1 (Monocyte chemoattractant protein 1), TNF-α, IL-6, lipid profiles, and boosted the synthesis of insulin and elevated activities of SOD and GSH-Px in STZ induced diabetic animals [124,125,130,131]. Taken together, polysaccharides obtained from various dietary sources can effectively ameliorate hyperglycemia, hyperlipidemia, low-grade inflammation, and oxidative stress in T2DM, and, therefore, intake of polysaccharides can be a potential beneficial choice for diabetes.

### 3.6. Inhibition of α-Amylase and α-Glucosidase

Generally, an experimental indicator of T2DM is hyperglycemia; it is well-defined as abnormally elevated fasting and postprandial glucose levels in the blood. Hence, managing postprandial hyperglycemia is a main beneficial strategy for the management of diabetes. Dietary carbohydrates are naturally digested into monosaccharides, such as glucose and fructose; these monosaccharides can be readily uptaken by the small intestine and transfer into the blood circulation. The human body normally has several dynamic carbohydrates–digestive enzymes, of which α-amylase (saliva or pancreas) and α-glucosidase (small-intestine) are most distinct. α-amylases are present in saliva and pancreas that degrade polysaccharide into glucose. Likewise, α-glucosidases are vital for assimilating oligosaccharides to monosaccharides in the small intestine. Hence, restraining of these digestive enzymes notably inhibit the conversion of polysaccharides into blood glucose, which serves as an effective step to control the blood glucose in diabetic patients [132]. In addition, the hypoglycemic effect of polysaccharides has been achieved by changing the small intestine transit time and preventing the carbohydrate digestion by suppression of digestive enzyme. These inhibitions are generally accomplished by dietary components, such as inulin, tannin, and phytic acid [133]. All these steps can be an effective strategy in diabetes for controlling the blood glucose level.

Various in vitro studies showed that low-molecular-weight bioactive polysaccharides obtained from the fruits of blackcurrant (Ribes nigrum L.), an alkaline soluble polysaccharide from *Coreopsis tinctoria*; fucoidan polysaccharide from *Turbinaria conoides*; polysaccharide fraction from *Diaphragma juglandis fructus* exhibited higher antioxidant, α-amylase, and α-glucosidase inhibitory activities that showed higher bioactive, with hypoglycemic, potential [134,135,136,137]. Furthermore, these polysaccharides significantly prevented the synthesis of NO, TNF-α, and IL-6 in LPS (Lipopolysaccharide)-stimulated BV2 (raf/myc-immortalised murine neonatal microglial cell line) microglial cells [134]. Hence, all these investigations recommended that bioactive polysaccharides could serve as potential hypoglycemic agents to be applied as functional foods or alternative supplements. Various molecular weight polysaccharides isolated from the pulp of apricot (*Armeniaca sibirica* L. Lam., MW: 25.93 kDa) [138]; seeds of *Plantago asiatica L*. (MW: 1894 kDa) [139]; Fucoidan from sea cucumber [67], *Turbinaria ornate* [140], *Fucus vesiculosus* [17], and *Sargassum wightii* [141] demonstrated significant inhibition of α-glucosidase and α-amylase activities in vitro. All these crude polysaccharide extracts exhibited a significant α-amylase and α-glucosidase inhibitory effect in a dose-dependent manner. Based on the observation in in vitro studies, all these results strongly suggest that polysaccharides remarkably inhibit carbohydrate digesting enzymes, α-glucosidase, and α-amylase activities, which regulate blood glucose levels. Hence, polysaccharides serve as an effective component to control the hyperglycemic conditions in diabetic patients.

### 3.7. Increasing Insulin Signaling Pathways

**Figure 2:**
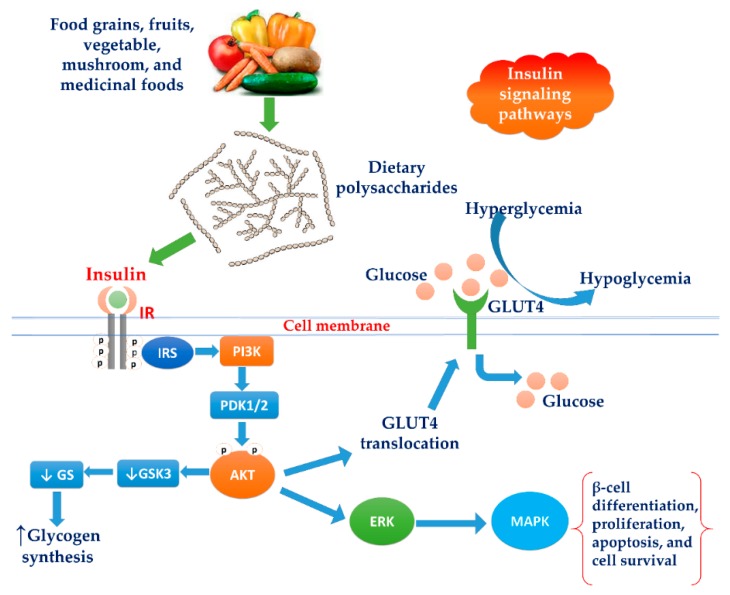
Schematic representation of the action of dietary polysaccharides on the insulin signaling pathway. Dietary polysaccharides elevate the cascade signaling of IRS/PI3K/AKT/GLUT4 and inhibit GSK3/GS, eventually stimulating the synthesis of glycogen. Akt is a main mediator to activate ERK/MAPK pathways and stimulate various physiological and biochemical mechanisms, including cell differentiation, proliferation, apoptosis, and cell endurance. IRS: insulin receptor substrate, PI3K: phosphoinositide 3-kinase, PDK1/2: Phosphoinositide-dependent protein kinase 1 and 2, AKT: serine/threonine-specific protein kinase, GSK-3: glycogen synthase kinase-3, GS: glycogen synthase, GLUT4: glucose transporter type 4, ERK: extracellular-signal-regulated kinase, MAPK: mitogen-activated protein kinase.

Elevated blood glucose normally elicits the synthesis of insulin in the pancreatic *β*-cells. Secretion of insulin instantly binds to its membrane receptor, which stimulates a cascade sequence of mechanism. This series of mechanism subsequently aids to increase glucose influx and metabolic effects, including glycolysis, glycogenesis, and avert glycogenolysis. Furthermore, insulin triggers regular cellular and physiological functions, comprising the cell division, apoptosis, and autophagy [3,142]. This cascade mechanism starts with the autophosphorylation of tyrosine residues in the intracellular components of the insulin receptor, which phosphorylate various substrates, including IRS1 and IRS2. Both substrates fix and activate the PI3K (PI3K: phosphoinositide 3-kinase)/Akt pathway as well as the MAPK (MAPK: mitogen-activated protein kinase) pathway. Akt is a main mediator to activate the most biochemical mechanism in glucose metabolism via activating phosphofructokinase and deactivating glycogen synthase kinase, resulting in stimulation of glucose transporter system translocation [3]. MAPK is a specific protein kinase involved in various physiological and biochemical mechanisms, including cell differentiation, proliferation, apoptosis, and cell endurance. ERK1/2 (extracellular-signal-regulated kinase 1/2) and JNK (c-Jun N-terminal kinase) are other cell signaling kinases co-task with MAPK, involved in cell growth, differentiation, inflammatory response, and apoptosis [4] (Figure 2). Overstimulation of MAPK generally provides the failure of insulin synthesis linked with apoptosis process in pancreatic islet cells [3].

#### 3.7.1. Activation of the PI3K/Akt Pathway

The different molecular weight of polysaccharides acquired from *Ophiopogon japonicas* (MW: 3.47, 6.746, 35.2, 124.3, and 324.6 kDa), *Acaudina molpadioides* (MW: 1614.1 kDa), and mulberry leaf (MW: 289) reduced hyperglycemia and hyperinsulinemia in STZ-induced diabetic mice. Polysaccharide triggers the PI3K/Akt signaling pathway through IRS1, PI3K-p85, and phosphorylated Akt develops insulin sensitivity [26,123,143] and improves diabetic-associated renal disease [144]. Moreover, treatment of these polysaccharides increased GLUT4 levels in pancreas and decreased glycogen synthase kinase-3*β* levels in most of the cells. This observation showed that polysaccharides demonstrated anti-diabetic agents by triggering the signaling pathways of PI3K/Akt/GSK-3/GLUT-4 [144].

Polysaccharide derived from *Ganoderma atrum, Enteromorpha prolifera,* and *Liriope spicata var.* prolifera (MW: 3.2 and 4.29 kDa) markedly decreased FBG and significantly improved plasma lipid profiles and glucose tolerance in diabetes-induced endothelial dysfunction in animal models. In addition, administration of polysaccharides remarkably inhibited the expression of GSK-3*β *(glycogen synthase kinase-3*β*) and elevated expressions of the insulin receptor, IRS1, PI3K, AKT, eNOS, and GLUT4 in the liver of diabetic rats [85,99,145,146,147]. An in vitro study also showed that the polysaccharide *from Grifola frondosa* significantly increased glucose metabolism and glycogen synthesis in HepG2 cells. Western blot findings demonstrated that polysaccharide triggered insulin receptor and elevated Akt expression, thereby inhibiting GSK-3*β* expression [148]. Another in vitro study demonstrated that the fruit of high molecular weight polysaccharide *Lycium barbarum* L. (MW: 33.867 kDa) elevated expressions of PI3K, p38 MAPK, and glucose uptake by GLUT4 in isolated adipocytes and reduced insulin receptors in obese and diabetic rats [74,149].

Sea cucumber containing polysaccharides consisted of a chondroitin sulfate E backbone, which decreased the level of glucose in the blood by stimulating PI3K/GLUT4 and elevated the phosphorylation of insulin receptors, IRS1, and p85-PI3K in the skeletal muscles of T2DM [90,150,151]. Western blot findings demonstrated that polysaccharides enhanced the protein expressions of IRS2, PI3K, and glycogen synthase and lowered that of GSK-3*β* in the liver of type 2 diabetic mice. At the end of the experiment, these findings suggested that sea cucumber polysaccharides increased glucose metabolism through the PI3K/GLUT4/GSK-3*β* signaling pathway [90,150,151]. A low molecular weight polysaccharide (120 kDa) extracted from tea (*Camellia sinensis* L.) demonstrated hypoglycemic, hypolipidemic, and insulin-sensitizing effects in obese and diabetic mice. In addition, these polysaccharides improved SOD, CAT, and GSH-Px activities in liver and kidney tissue of diabetic mice. Tea polysaccharides increased the expressions of PI3K/AKT p-AKT and GLUT4 signaling pathway [27,88,152]. All these above findings proved that administration of polysaccharides remarkably inhibited the expression of GSK-3*β* and elevated expressions of the insulin receptor, IRS1, PI3K, and AKT in type 2 diabetic animal models.

#### 3.7.2. Modulation of the MAPK Pathway

Sea cucumber polysaccharide (MW: 21.53 kDa) purified from *Acaudina Molpadioides* (MW: 20.53 kDa,) and pumpkin polysaccharides (MW: 749.3, 727.0, and 607.6 kDa) and sulfated rhamnose polysaccharides (MW: 4.57 kDa) from *Enteromorpha prolifera* increased insulin-stimulated glucose uptake, GLUT4 translocation, and Akt/ERK activation in TNF-α-induced insulin-resistant 3T3-L1 adipocytes. This finding strongly suggested that polysaccharide enhanced glucose uptake by activating the PI3K/Akt pathway and MAPK–ERK pathway [90,150,151,152]. Another in vitro study connected with active polysaccharides on LPS-induced RAW 264.7 cells; polysaccharides derived from *Agaricus blazei* Murill decreased the expression of JNK, ERK, and p38 [132,153,154]. Xu et al. [155] purified polysaccharide (MW: 460 kDa) from *Ramulus mori* (*M. alba* L.) that decreased FBG and HbA1c levels and augmented insulin levels in STZ-induced type 2 diabetic mice. Western blot studies in pancreatic tissue exhibited that polysaccharide down-regulated the expression of p-JNK, p-p38, Bax, and cleaved-caspase-3 and increased Bcl-2 expression. This study strongly suggested that polysaccharide had a hypoglycemic effect by down-regulating the JNK/p38 pathway to inhibit pancreatic cell apoptosis [155].

## 4. Conclusions

Diabetes is currently a serious health issue worldwide producing significant morbidity and mortality, and there is no route to cure diabetes completely. The commercial oral hypoglycemic and anti-hyperglycemic drugs have their self-limitations, adverse effects, high cost, and secondary failure. In addition, these oral diabetic drugs cause serious complications, such as hypoglycemia, weight gain, abdominal pain, nausea, vomiting, edema, diarrhea, gas trouble, bloating, and an increase levels of LDL-C. Hence, screening active anti-diabetic agents from natural sources, including polysaccharide, is of greater attraction due to its lesser side effects. Various in vivo, in vitro, and clinical experiments in this review clearly showed that oral administration of polysaccharides reduced hyperglycemia and hyperlipidemia through underlying various molecular mechanisms. Anti-diabetic effects are mediated primarily by their antioxidant properties, as well as the succeeding methods include inhibition of α-amylase and α-glucosidase activity, improving glucose metabolism, increasing *β*-cell mass, and reducing *β*-cell dysfunction. Insulin signaling pathways are also increased through activating PI3K/Akt pathway and modulating MAPK/JNK/ERK pathway. Hypercholesterolemic and hyperlipidemic activities are also associated with diabetes in which dietary polysaccharides have a vital function in the activation of AMPK pathway and down-regulation of ACC, SREBP-1c, and HMG-CoA reductase that leads to reduce the levels of triglycerides and cholesterol. Hence, dietary polysaccharides could be considered as anti-diabetic agents and involved in alleviating the pathogenesis, leading to reduce the degree of *β*-cell damage in the pancreas.

## Figures and Tables

**Table 1:** In vitro and in vivo actions of dietary polysaccharides and their anti-diabetic potential.

Source of Polysaccharides	Botanical Name/Composition	Model	Doses and Route of Administration	Negative Control	Investigation	Results	References
Mushroom	*Cordyceps militaris*	Wistar mice	100 and 400 mg/kg, p.o. for 4 weeks	STZ (60 mg/kg, i.p)	FBG, Serum Insulin, OGTT, AST, ALT, BUN, CRE, LDL-C, TC, HDL-C, hepatic, renal, and pancreatic SOD, GSH-Px, CAT, and lipid peroxidation	Antioxidant and hypoglycemic effects	[23]
Mushroom	*Cordyceps sinensis, Omphalia lapidescens, and Tricholomamongolicum*	Wistar rats	10 and 100 mg/kg, p.o. for 4 weeks	STZ (40 mg/kg, i.p)	FBG and PBG	Antioxidant and hypoglycemic effects	[24]
Mushroom	*Cordyceps sinensis, Omphalia lapidescens,* and *Tricholomamongolicum.*	SD male rats	500 mg and 2000 mg/kg, p.o. for 3 weeks	STZ (40 mg/kg, i.p)	FBG, PK, SOD, GSH-Px, TG, TC, BUN, UA, CRE, and urine protein levels	Anti-diabetic and anti-nephropathic activities	[25]
Mushroom	*Cordyceps militaris*	Sprague-Dawley male rats	0.5, 1.0, and 2.0 g/kg, p.o. for 4 weeks	STZ (40 mg/kg, i.p)	FBG, PK, SOD, GSH-Px, TG, TC, BUN, UA, CRE, urine protein, NAG, and MDA	Anti-diabetic and antinephritic activities	[26]
Mushroom	*Cordyceps mycelia*	Male BALB/c mice and male Sprague-Dawley rats	200 mg, 400 mg/kg, p.o. for 1-week	STZ (60 mg/kg, i.p), Alloxan monohydrate (150 mg/kg, i.p.)	Blood glucose and insulin	Hypoglycemic activity	[27]
Mushroom	*Cordyceps militaris*	C57BL/6J mice	360 mg/kg/p.o. for 8 weeks	HFD + STZ (60 mg/kg, i.p) + nicotinamide (180 mg/kg, i.p)	FBG, OGTT, IPITT, CRE, AGEs, TGF-*β*1, TC, TG, LDL-C, and HDL-C	Anti-diabetic and renoprotective activities	[28]
Mushroom	*Paecilomyces hepiali*	Sprague-Dawley male rats	0.08, 0.4, and 2.0 g/kg/p.o. for 4 weeks	HFD + STZ (25 mg/kg, i.p) + nicotinamide (180 mg/kg, i.p)	Blood glucose, TC, LDL-C, insulin, PK, glycogen, SOD, MDA, GSH-Px IL-2, IL-6, IL-10, and TNF-α	Anti-diabetic and antinephritic Activities	[29]
Mushroom	*Inonotus obliquus*	HepG2 cells and insulin-resistant HepG2 cells	10, 20, 40, 80, and 160 μg/mL, for 24 and 48 h.	-	Glucose, insulin	Hypoglycemic activity	[30]
Mushroom	*Antrodia cinnamomea*	-	50 μL	-	α-glucosidase inhibitory activity	Anti-diabetic activity	[31]
Mushroom	*Grifola frondosa*	Male ICR mice, HepG2	75 and 150 mg/kg for 0, 14, and 28 days; 100 μg/mL	STZ (40 mg/kg, i.p)	Glucose, OGTT, insulin, IRS1, JNK1, PI3K, or GLUT4	Anti-diabetic activity	[15,32]
Mushroom	*Aronia melanocarpa*, red ginseng, and shiitake mushroom	Male SD rats	0.5, 1 g/kg bw	Pancreatectomy rats with 1 g dextrin/kg bw	Serum glucose, food intake, body weight, and OGTT	Anti-diabetic activity	[33]
Mushroom	*Chroogomphus rutilus*	Male SD rats	1.0 and 2.0 g/kg bw, p.o. for 4 weeks	STZ (40 mg/kg, i.p)	α-glucosidase, blood glucose, SOD, GSH-Px, MDA, TC, TG, LDL-C, HDL-C, and MTT	Antioxidant, Hypoglycemic, Hypolipidemic, and Antitumor Activities	[34]
Mushroom	*Lignosus rhinocerotis*	Male SD rats	0.5, 1.0, and 2.0 g/kg bw, p.o. for 8 weeks	STZ (35 mg/kg, i.v.)	Blood glucose, GSH, CAT, SOD, and LPO	Anti-diabetic activity	[35]
Mushroom	*Agaricus brasiliensis and Ganoderma lucidum*	Male SD rats	1.0 and 2.0 g/kg bw, p.o. for 4 weeks	STZ (35 mg/kg, i.v.)	Blood glucose, GSH, CAT, SOD, LPO, TBARS, GSH-Px, and GSH-R	Anti-diabetic activity	[36]
Mushroom	*Pleurotus Ostreatus*	KK-A^y^ Mice	1.0 and 2.0 g/kg bw, p.o. for 4 weeks	STZ (35 mg/kg, i.v.)	Blood glucose, AMPK, GLUT-4, Akt, and PKC	Anti-diabetic activity	[37]
Mushroom	*Pleurotus Ostreatus*	Rabbits	100, 200, and 300 mg/kg for 4 weeks	Alloxan (120 mg/kg, p.o)	Blood glucose, ALP, γGT, ALT, AST, bilirubin, urea, BUN, CRE, Na, and K	Anti-diabetic activity	[38]
Mushroom	*Inonotus obliquus*	Male Kunming mic	900 mg/kg for 4 weeks	STZ (60 mg/kg, i.p.)	Blood glucose, body weight, organ weight, glycogen, OGTT, TC, TG, LDL-C, HDL-C, PI3K, GLUT-4, and Akt	Anti-diabetic activity	[39]
Mushroom	*Pleurotus citrinopileatus*	In vitro	-	-	Pancreatic α-amylase, intestinal α-glucosidase, and ACE	Antioxidant, Hypoglycemic and Hypotensive Activities	[40]
Mushroom	*Catathelasma ventricosum*	Male ICR mice	0.2 g/kg for 4 weeks	STZ (150 mg/kg, i.p.)	Blood glucose, TC, TG, LDL-C, and HDL-C	Anti-diabetic activity	[41]
Mushroom	*Pleurotus ostreatus, Calocybe indica, and Volvariella volvacea*	In vitro, in vivo (Male ICR mice)	200 and 400 mg/kg for 6 weeks	STZ (150 mg/kg, i.p.)	α-amylase inhibition assay, glucose uptake by yeast cells, glucose adsorption capacity, and blood glucose	Anti-diabetic activity	[42]
Mushroom	*Pleurotus eryngii*	KKAy mice	1 g/kg for 6 weeks	STZ (150 mg/kg, i.p.)	Blood glucose, insulin, FBS, OGTT, TC, TG, LDL-C, HDL-C, liver glycogen	Hypolipidemic and hypoglycemic activities	[43]
Grains	Foxtail Millet	Open-label, self-controlled clinical trial 64 subjects (27 male subjects and 37 female subjects)	50–150 g of whole grain for week 6 and 12	Diabetic patients	FBG, insulin, fructosamine, fasting C-peptide, TG, and TC HDL-C, LDL-C, apolipoprotein A1 and B, TNF-α, IL-6, leptin, GLP-1, blood pressure, body weight, waist circumference, and hip circumference	Anti-diabetic activity	[44]
Vegetable, fruit, and grain	Vegetable, fruit, and grain	48,835 post-menopausal women	A 1:1:0.5–serving/day vegetable, fruit, food grains	Diabetic patients	Serum glucose, insulin, and waist circumference	Reduced the risk of diabetes	[45]
Whole Grain cereals	Whole grain cereals	A meta-analysis of randomized controlled trials	50 g/day	Healthy Subjects	Serum glucose, insulin, and HbAlc	Improved the PBG and insulin homeostasis	[46]
Grain and Sprouted grain	Grain and sprouted grain	12 male subjects	50 g/day	Healthy Subjects	Serum glucose, insulin, and HbAlc	Only sprouted-grain improved PBG and insulin	[47]
Whole Grains muffins	Wheat, rice, corn, oat, and barley	4 Male and 8 Female	50 g/day	Healthy Subjects	Serum glucose, insulin, and HbAlc	Lowered the PBG	[48]
Whole grains bread	Chickpea-wheat composite bread	13 female subjects	50 g/day	Healthy Subjects	Serum glucose, insulin, and HbAlc	Reduced PBG	[49]
Whole grains bread	Maize	30 male subjects	50 g/day	Healthy Subjects	Serum glucose	Reduced PBG	[50]
Sorghum and Wheat muffin	Sorghum and wheat flour	10 male subjects	50 g/day	Healthy Subjects	Serum glucose, insulin	Improved the PBG and insulin	[51]
Whole rye bread	Whole rye with white wheat bread	6 males and 9 females	50 g/day	Healthy Subjects	Serum glucose, insulin	Improved the insulin response	[52]
Oat	Oat	A meta-analysis of randomized controlled trials	50 g/day	Healthy Subjects	Serum glucose, insulin	Improved glucose and insulin response	[53]
Oat and beta-glucan	Oat and beta-glucan	A meta-analysis of randomized controlled trials	-	Healthy Subjects	Serum glucose, HbA1c, and insulin	Improved glucose and insulin and HbA1c response	[54]
Whole grain rye with starch	Whole grain rye flour and rye kernels bread	21 subjects	50 g/day	Healthy Subjects	Serum glucose, OGTT, insulin, PYY, FFA, and IL-6	Improved cardiometabolic variables and glucose	[55]
Whole grain oats	Whole grain oats	A meta-analysis of randomized controlled trials	-	Healthy Subjects	Serum glucose, OGTT, insulin, and TC	Cholesterol-lowering and anti-diabetic effects	[56]
Whole-grain rye and wheat bread	Whole-grain rye porridges and refined wheat bread	21 subjects	40, 55 g/day	Healthy Subjects	Serum glucose, postprandial plasma amino acids and short chain fatty acids	Suppressed appetite and improved glucose metabolism.	[57]
Canola oil-enriched bread supplement	Canola oil-enriched bread	141 subjects	31 g/day	Diabetic patients	HbA1c, blood pressure, Framingham CVD risk score, and reactive hyperemia index ratio	Improved glycemic control in T2DM	[58]
Grains	Monascus-fermented grains	Male SD rats	300 mg/kg bw. For 16 weeks	High-fructose (60%, *w*/*w*) plus high-fat (20%, *w*/*w*) diet	OGTT, Insulin, insulin sensitivity index, TBARS, SOD, CAT, and GPx	Anti-diabetic effect by improving insulin resistance and hepatic antioxidant enzymes.	[59]
Whole grains and legumes	Whole grains and legumes	39 males, 146 females	30–70 g for 16 weeks	Diabetic patients	BMI, waist and hip ratio, TC, TG, LDL-C, HDL-C, FBS, insulin FFA, Plasma apolipoprotein A-V, and CRP	Anti-diabetic effects	[60]
DASH diet	fruits, vegetables, whole grains, low-fat dairy products, low in saturated fats, cholesterol, refined grains, and sweets	52 pregnant women	40 g for 4 weeks	Gestational Diabetic patients	Length, weight, and head circumference of infants	Improved gestational diabetes mellitus	[61]
Whole grains	Cereal, bread, rice, pasta, and muffin	11 subjects	6–10 servings/day for 6 weeks	Diabetic/obese patients	Insulin, blood glucose, and OGTT	Reduce the risk of T2DM and heart disease.	[62]
Vegetables	Okra (*Abelmoschus esculentus* L. Moench)	Male C57BL/6 mice	50 mg/kg, p.o for 10 days	STZ (45 mg/kg, i.p.)	blood glucose, OGTT	Hypoglycemic effect	[63]
Vegetables	Red pepper and soybeans	Male SD rats	5% powder supplement	STZ (45 mg/kg, i.p.)	FBS, OGTT, body weight, visceral fat, and serum leptin	Improves glucose homeostasis by reducing insulin resistance	[64]
Fruits and vegetables	Fruits and vegetables	550 children and adolescents	257, 227 g/day for 30 days	Diabetic patients	FBS, insulin, and HbA1c	Anti-diabetic effect	[50]
Vegetables	Purple carrots and purple potatoes	Obese Zucker rats	Purple carrot and potatoes supplemented a high-fat diet for 8 weeks.	-	Intraperitoneal glucose and insulin tolerance test and invasive hemodynamic tests	Purple vegetables improve insulin resistance and hypertension	[65]
Apricot Lychee	*Prunus armeniaca Lychee chinensis*	In vitro	-	-	α-glycosidase, aldose reductase, and antioxidant activity	Anti-diabetic effects	[66]
Blueberry	*Vaccinium cyanococcus*						
Plum	*Prunus salicina*						
Kiwi	*Kiwifruit c.v. hayward*						
Lemon pulp	*Citrus limon*						
Lemon peel	*Citrus limon*						
Pear	*Pyrus bretschneider*						
Wolfberry	*Lycium chinensis*						
Watermelon	*Citrullus lanatusus*						
Lettuce	*Lactuca sativa*	In vitro	-	-	α-glycosidase, aldose reductase, and antioxidant activity	Anti-diabetic effects	[66]
Cucumber	*Cucumis sativus*						
Red onion	*Allium cepa*						
Bitter gourd	*Momordica charantia*						
Eggplant	*Solanum melongena*						
Celery	*Apium graveolens*						
Kelp	*Laminaria japonica*						
Wax gourd	*Benincasa pruriens*						
Garlic	*Allium sativum*						
Tomato	*Solanum lycopeersicum*						
Vegetables	*Momordica charantia*	SD rats	50 mg/kg, p.o for 10 days	STZ (45 mg/kg, i.p.)	FBS, insulin, and HbA1c	Anti-diabetic effects	[67]

**Abbreviations:** ACE-angiotensin converting enzyme; AGEs-advanced glycation end products; Akt-serine/threonine-specific protein kinase; ALP-alkaline phosphatase; ALT-alanine transaminase; AST-aspartate transaminase; BUN-blood urea nitrogen; CAT-catalase; CRE-creatinine; CRP-C-reactive protein; FBG-fasting blood glucose; FFA-free fatty acids; GLP-1-glucagon-like peptide-1; GLUT4-glucose transporter 4; GSH-Px-glutathione peroxidase; GSH-R-glutathione reductase; HbA1c-glycated hemoglobin; HDL-C-high density lipoprotein–C; HepG2-human liver cancer cell line; HFD-high-fat diet; IL-interleukin; IL-6-interleukin-6; IPITT-intraperitoneal insulin tolerance test; IRS1-insulin receptor substrate 1; JNK-c-Jun N-terminal kinases; K-potassium; LDL-C-low density lipoprotein–C; LPO-lipid peroxidation; MDA-malondialdehyde; MTT-3-(4,5-dimethylthiazol-2-yl)-2,5-diphenyltetrazolium; Na-sodium; NAG-n-acetyl-*β*-d-glucosaminidase; OGTT-oral glucose tolerance test; PBG-postprandial glucose; PI3K-phosphoinositide 3-kinases; PK-pyruvate kinase; PKC-protein kinase *C*; PYY-peptide tyrosine tyrosine hormone; SD-Sprague-Dawley; SOD-superoxide dismutase; TC-total cholesterol; T2DM-type 2 diabetes; TG-triglycerides; TGF-*β*1-transforming growth factor-*β*1; TNF-α-tumor necrosis factor-α; UA-uric acid; γGT-gamma-glutamyltransferase; i.v-intravenous; i.p-intraperitoneal; b.w-body weight; DASH-dietary approaches to stop hypertension.

**Table 2:** Anti-cholesterolemic and anti-triglyceridemic effects of dietary polysaccharides.

Sources of Polysaccharides	Monosaccharide Units/Active Compounds	Effects on Metabolism	Molecular Mechanisms	Results	References
*Cyclocarya paliurus*	Rhamnose, arabinose, xylose, mannose, glucose, and galactose	Triglyceride metabolism	↑ATGL, ↑PPAR-α, ↑PPARɣ coactivator-1 α, ↓FAS, ↓HMG-CoA reductase	Anti-hyperlipidemic effects	[103]
*Cichorium intybus* L.	Sorbin, glucose, fructose, and glucitol	Triglyceride metabolism	↑p-AMPK, ↑ATGL, ↑CAPT1, ↑p-ACC, ↓FAS,	Anti-hyperlipidemic effects	[104]
*Lycium barbarum*	Rhamnose, arabinose, xylose, mannose, glucose, galactose, and galacturonic acid	Triglyceride metabolism	↑p-AMPK, ↑p-ACC, ↑ATGL, ↑CAPT1, ↓FAS	Anti-hyperlipidemic effects	[105]
*Enteromorpha prolifera*	Rhamnose, glucuronic acid, arabinose, fucose, xylose, and glucose	Cholesterol metabolism	↓SREBP-2, ↓HMG-CoA reductase	Cholesterol-lowering effects	[106]
*Oryza sativa* L.	Xylose, rhamnose, mannose, galactose, arabinose, and glucose	Triglyceride and cholesterol metabolism	↑PPAR-α, ↑PPARɣ coactivator-1 α, ↓SREBP-1c	Anti-hyperlipidemic effects	[107]
*Morchella angusticepes*	Arabinose, mannose, glucose, and galactose	Cholesterol metabolism	↓HMG-CoA reductase	Cholesterol-lowering effects	[109]
*Lentinula edodes*	α- and *β*-glucans and fucomannogalactans	Cholesterol metabolism	↓HMG-CoA reductase	Cholesterol-lowering effects	[110]
*Fucus vesiculosus*	Sulfated polysaccharide with fucose	Triglycerides and cholesterol metabolism	↓FAS, ↓ACC, ↓SREBP -1c, ↓SREBP-2, ↓HMG-CoA reductase	Triglyceride and Cholesterol-lowering effects	[111]
*Lycium barbarum*	Rhamnose, arabinose, xylose, mannose, glucose, galactose, and galacturonic acid	Triglyceride and cholesterol metabolism	↑p-AMPK, ↑PPARɣ coactivator-1 α, ↑p-ACC, ↓FAS, ↓SREBP-1c	Anti-hyperlipidemic effects	[116]
*Rheum palmatum* L.	Rhamnose, mannose, and galactose	Triglyceride metabolism	↑p-AMPK, ↑p-ACC	Anti-hyperlipidemic effects	[117]
*Schisandra Chinensis*	Galactose, arabinose, and glucose	Triglyceride and cholesterol metabolism	↓SREBP-1c, ↓SREBP-2, ↓FAS ↓ACC, ↓HMG-CoA reductase	Anti-hyperlipidemic effects	[118]
*Aconiti* Lateralis *Radix Praeparata*	α-d-glucan	Cholesterol metabolism	↑LDL receptor, ↓HMG-CoA reductase	Cholesterol-lowering effects	[119]
*Brasenia schreberi*	Galactose, mannose, fucose, rhamnose, arabinose, xylose, glucose, and alduronic acids	Cholesterol metabolism	↑LDL receptor, ↑PPAR-α	Cholesterol-lowering effects	[120]

**Abbreviations:** ATGL-adipose triglyceride lipase; PPAR-α-peroxisome proliferator-activated receptor alpha; FAS-fatty acid synthase; HMG-CoA reductase-3-hydroxy-3-methylglutaryl-CoA reductase; p-AMPK-phosphorylated serine/threonine protein kinase; CAPT1-carnitine palmitoyltransferase-1; p-ACC-phosphorylated acetyl-CoA carboxylase; SREBP-2-sterol regulatory element binding protein-2; SREBP-1c-sterol regulatory element binding protein-1c, ↑ increase; ↓ decrease.

## Abbreviations

ACC	acetyl-CoA carboxylase
ACE	angiotensin converting enzyme
AGEs	advanced glycation end products
Akt	serine/threonine-specific protein kinase
ALP	alkaline phosphatase
ALT	alanine transaminase
AMPK	serine/threonine protein kinase
AST	aspartate transaminase
Bax	BCL2 associated X protein
Bcl-2	B-cell lymphoma 2
BUN	blood urea nitrogen
CAT	catalase
CPT1	carnitine palmitoyltransferase-1
CRE	creatinine
CVD	cardiovascular diseases
CRP	c-reactive protein
eNOS	endothelial nitric oxide synthase
ERK	extracellular-signal-regulated kinase
FBG	fasting blood glucose
FFA	free fatty acids
GLP-1	glucagon-like peptide-1
GLUT4	glucose transporter 4
GSH-Px	glutathione peroxidase
GSH-R	glutathione reductase
GSK- 3	glycogen synthase kinase-3
HbA1c	glycated hemoglobin
HDL-C	high-density lipoprotein –C
HepG2	human liver cancer cell line
HFD	high-fat diet
IF-γ	interferon γ
IL	interleukin
IL-6	interleukin-6
InsR	insulin receptor
IPITT	Intraperitoneal Insulin Tolerance Test
IRS1	Insulin receptor substrate 1
IRS-1,2	insulin receptor-1,2
JNK	c-Jun N-terminal kinases
K	potassium
KDa	kilodaltons
LDL-C	low-density lipoprotein -C
LPO	lipid peroxidation
LPS	lipopolysaccharides
MAPK	mitogen-activated protein kinases
MCP-1	Monocyte chemoattractant protein-1
MDA	malondialdehyde
mRNA	messenger RNA
MTT	3-(4,5-Dimethylthiazol-2-yl)-2,5-diphenyltetrazolium
Na	sodium
NAG	n-acetyl-*β*-d-glucosaminidase
NO	nitric oxide
OGTT	oral glucose tolerance test
OGTT	oral glucose tolerance test
PBG	postprandial glucose
PDX-1	insulin promoter factor 1
PI3K	Phosphoinositide 3-kinases
PK	pyruvate kinase
PKC	protein kinase *C*
PPAR-α	peroxisome proliferator-activated receptor-alpha
PYY	peptide YY hormone
SD	Sprague-Dawley
SOD	superoxide dismutase
SREBP-1c	sterol regulatory element binding protein -1c
SREBP-2	sterol regulatory element binding protein -2
STZ	streptozotocin
T2DM	T2DM
TC	total cholesterol
TG	triglycerides
TGF-*β*1	transforming growth factor-*β*1
TNF-α	tumor necrosis factor-α
UA	uric acid
γGT	gamma-glutamyltransferase

## References

[1] Ganesan K., Chung S.K., Vanamala J., Xu B. (2018). Causal relationship between diet-induced gut microbiota changes and diabetes: A novel strategy to transplant *Faecalibacterium prausnitzii* in preventing diabetes. Int. J. Mol. Sci..

[2] Kumar G., Sharmila Banu G., Murugesan A.G. (2009). Attenuation of *Helicteres isora* L. bark extracts on streptozotocin-induced alterations in glycogen and carbohydrate metabolism in albino rats. Hum. Exp. Toxicol..

[3] Jayachandran M., Zhang T., Ganesan K., Xu B., Chung S.S.M. (2018). Isoquercetin ameliorates hyperglycemia and regulates key enzymes of glucose metabolism via insulin signaling pathway in streptozotocin-induced diabetic rats. Eur. J. Pharmacol..

[4] Jayachandran M., Wu Z., Ganesan K., Khalid S., Chung S.M., Xu B. (2019). Isoquercetin upregulates antioxidant genes, suppresses inflammatory cytokines and regulates AMPK pathway in streptozotocin-induced diabetic rats. Chem. Biol. Interact..

[5] Sukalingam K., Ganesan K., Ponnusamy K. (2015). Evaluation of antidiabetic activity of polyherbal formulations on type 2 diabetic patients: A single blinded randomized study. Int. J. Integ. Med. Sci..

[6] Ganesan K., Jayachandran M., Xu B. (2017). A critical review on hepatoprotective effects of bioactive food components. Crit. Rev. Food Sci. Nutr..

[7] Ganesan K., Xu B. (2017). Molecular targets of vitexin and isovitexin in cancer therapy: A critical review. Ann. N. Y. Acad. Sci..

[8] Ganesan K., Xu B. (2017). Polyphenol-rich dry common beans (*Phaseolus vulgaris* L.) and their health benefits. Int. J. Mol. Sci..

[9] Ganesan K., Xu B. (2017). Polyphenol-rich lentils and their health promoting effects. Int. J. Mol. Sci..

[10] Wang D., Li C., Fan W., Yi T., Wei A., Ma Y. (2019). Hypoglycemic and hypolipidemic effects of a polysaccharide from Fructus Corni in streptozotocin-induced diabetic rats. Int. J. Biol. Macromol..

[11] Xiao H., Chen C., Li C., Huang Q., Fu X. (2019). Physicochemical characterization, antioxidant and hypoglycemic activities of selenized polysaccharides from *Sargassum pallidum*. Int. J. Biol. Macromol..

[12] Lu A., Yu M., Fang Z., Xiao B., Guo L., Wang W., Li J., Wang S., Zhang Y. (2019). Preparation of the controlled acid hydrolysates from pumpkin polysaccharides and their antioxidant and antidiabetic evaluation. Int. J. Biol. Macromol..

[13] Zhao T., Mao G.-H., Zhang M., Li F., Zou Y., Zhou Y., Zheng W., Zheng D.-H., Yang L.-Q., Wu X.-Y. (2012). Anti-diabetic effects of polysaccharides from ethanol-insoluble residue of *Schisandra chinensis* (Turcz.) Baill on alloxan-induced diabetic mice. Chem. Res. Chin. Univ..

[14] Liu C., Song J., Teng M., Zheng X., Li X., Tian Y., Pan M., Li Y., Lee R.J., Wang D. (2016). Antidiabetic and antinephritic activities of aqueous extract of *Cordyceps militaris* fruit body in diet-streptozotocin-induced diabetic Sprague Dawley rats. Oxid. Med. Cell. Longev..

[15] Chen Y., Liu Y., Sarker M.M.R., Yan X., Yang C., Zhao L., Lv X., Liu B., Zhao C. (2018). Structural characterization and antidiabetic potential of a novel heteropolysaccharide from *Grifola frondosa* via IRS1/PI3K-JNK signaling pathways. Carbohydr. Polym..

[16] Tang H.-L., Chen C., Wang S.-K., Sun G.-J. (2015). Biochemical analysis and hypoglycemic activity of a polysaccharide isolated from the fruit of *Lycium barbarum* L.. Int. J. Biol. Macromol..

[17] Kim K.-T., Rioux L.-E., Turgeon S.L. (2015). Molecular weight and sulfate content modulate the inhibition of α-amylase by fucoidan relevant for type 2 diabetes management. Pharma. Nutr..

[18] Ye M., Qiu T., Peng W., Chen W.-X., Ye Y.-W., Lin Y.-R. (2011). Purification, characterization and hypoglycemic activity of extracellular polysaccharides from *Lachnum calyculiforme*. Carbohydr. Polym..

[19] Wang J., Jin W., Zhang W., Hou Y., Zhang H., Zhang Q. (2013). Hypoglycemic property of acidic polysaccharide extracted from *Saccharina japonica* and its potential mechanism. Carbohydr. Polym..

[20] Ma Y., Mao D., Geng L., Wang Z., Xu C. (2013). Production, fractionation, characterization of extracellular polysaccharide from a newly isolated *Trametes gibbosa* and its hypoglycemic activity. Carbohydr. Polym..

[21] Xu W., Zhou Q., Yin J.-J., Yao Y., Zhang J.-L. (2015). Anti-diabetic effects of polysaccharides from *Talinum triangulare* in streptozotocin (STZ)-induced type 2 diabetic male mice. Int. J. Biol. Macromol..

[22] Hong T., Zhao J., Dong M., Meng Y., Mu J., Yang Z. (2012). Composition and bioactivity of polysaccharides from *Inula britannica* flower. Int. J. Biol. Macromol..

[23] Zhao H., Lai Q., Zhang J., Huang C., Jia L. (2018). Antioxidant and hypoglycemic effects of acidic-extractable polysaccharides from *Cordyceps militaris* on type 2 diabetes mice. Oxid. Med. Cell. Longev..

[24] Zhang G., Huang Y., Bian Y., Wong J.H., Ng T.B., Wang H. (2006). Hypoglycemic activity of the fungi *Cordyceps militaris*, *Cordyceps sinensis*, *Tricholoma mongolicum*, and *Omphalia lapidescens* in streptozotocin-induced diabetic rats. Appl. Microbiol. Biotechnol..

[25] Dong Y., Jing T., Meng Q., Liu C., Hu S., Ma Y., Liu Y., Lu J., Cheng Y., Wang D. (2014). Studies on the antidiabetic activities of *Cordyceps militaris* extract in diet-streptozotocin-induced diabetic Sprague-Dawley rats. BioMed Res. Int..

[26] Liu Y., Chen D., You Y., Zeng S., Hu Y., Duan X., Liu A., Chen H., Hu X., Chen S. (2016). Structural characterization and antidiabetic activity of a glucopyranose-rich heteropolysaccharide from *Catathelasma ventricosum*. Carbohydr. Polym..

[27] Li S.P., Zhang G.H., Zeng Q., Huang Z.G., Wang Y.T., Dong T.T.X., Tsim K.W.K. (2006). Hypoglycemic activity of polysaccharide, with antioxidation, isolated from cultured *Cordyceps mycelia*. Phytomedicine.

[28] Yu S.-H., Dubey N.K., Li W.-S., Liu M.-C., Chiang H.-S., Leu S.-J., Shieh Y.-H., Tsai F.-C., Deng W.-P. (2016). *Cordyceps militaris* treatment preserves renal function in type 2 diabetic nephropathy mice. PLoS ONE.

[29] Wang J., Teng L., Liu Y., Hu W., Chen W., Hu X., Wang Y., Wang D. (2016). Studies on the antidiabetic and antinephritic activities of *Paecilomyces hepiali* water extract in diet-streptozotocin-induced diabetic Sprague Dawley rats. J. Diabet. Res..

[30] Xue J., Tong S., Wang Z., Liu P. (2018). Chemical characterization and hypoglycaemic activities *in vitro* of two polysaccharides from *Inonotus obliquus* by submerged culture. Molecules.

[31] Huang H., Wang S.-L., Nguyen V., Kuo Y.-H. (2018). Isolation and identification of potent antidiabetic compounds from *Antrodia cinnamomea*—An edible Taiwanese mushroom. Molecules.

[32] Chen X., Qian L., Wang B., Zhang Z., Liu H., Zhang Y., Liu J. (2019). Synergistic hypoglycemic effects of pumpkin polysaccharides and puerarin on type II diabetes mellitus mice. Molecules.

[33] Yang H., Kim M., Kwon D., Kim D., Zhang T., Ha C., Park S. (2018). Combination of aronia, red ginseng, shiitake mushroom and nattokinase potentiated insulin secretion and reduced insulin resistance with improving gut microbiome dysbiosis in insulin deficient type 2 diabetic rats. Nutrients.

[34] Zhang J., Zhao X., Zhao L.Q., Zhao J., Qi Z., Wang L.-A. (2017). A primary study of the antioxidant, hypoglycemic, hypolipidemic, and antitumor activities of ethanol extract of brown slimecap mushroom, *Chroogomphus rutilus* (Agaricomycetes). Int. J. Med. Mushrooms.

[35] Nyam K.L., Chow C.F., Tan C.S., Ng S.T. (2017). Antidiabetic properties of the Tiger’s Milk medicinal mushroom, *Lignosus rhinocerotis* (Agaricomycetes), in streptozotocin-induced diabetic rats. Int. J. Med. Mushrooms.

[36] Vitak T.Y., Wasser S.P., Nevo E., Sybirna N.O. (2017). Enzymatic system of antioxidant protection of erythrocytes in diabetic rats treated with medicinal mushrooms *Agaricus brasiliensis* and *Ganoderma lucidum* (Agaricomycetes). Int. J. Med. Mushrooms.

[37] Xiong M., Huang Y., Liu Y., Huang M., Song G., Ming Q., Ma X., Yang J., Deng S., Wen Y. (2018). Antidiabetic activity of ergosterol from *Pleurotus ostreatus* in KK-Ay mice with spontaneous type 2 diabetes mellitus. Mol. Nutr. Food Res..

[38] Ikewuchi C.C., Ikewuchi J.C., Ifeanacho M.O. (2017). Restoration of plasma markers of liver and kidney functions/integrity in alloxan-induced diabetic rabbits by aqueous extract of *Pleurotus tuberregium* sclerotia. Biomed. Pharmacother..

[39] Wang J., Wang C., Li S., Li W., Yuan G., Pan Y., Chen H. (2017). Anti-diabetic effects of *Inonotus obliquus* polysaccharides in streptozotocin-induced type 2 diabetic mice and potential mechanism via PI3K-Akt signal pathway. Biomed. Pharmacother..

[40] Chen P.-H., Weng Y.-M., Lin S.-M., Yu Z.-R., Wang B.-J. (2017). Molecular weight affected antioxidant, hypoglycemic and hypotensive activities of cold water extract from *Pleurotus citrinopileatus*. J. Food Sci..

[41] Liu Y., You Y., Li Y., Zhang L., Yin L., Shen Y., Li C., Chen H., Chen S., Hu B. (2017). The characterization, selenylation and antidiabetic activity of mycelial polysaccharides from *Catathelasma ventricosum*. Carbohydr. Polym..

[42] Singh V., Bedi G.K., Shri R. (2017). In vitro and in vivo antidiabetic evaluation of selected culinary-medicinal mushrooms (Agaricomycetes). Int. J. Med. Mushrooms.

[43] Chen L., Zhang Y., Sha O., Xu W., Wang S. (2016). Hypolipidaemic and hypoglycaemic activities of polysaccharide from *Pleurotus eryngii* in Kunming mice. Int. J. Biol. Macromol..

[44] Ren X., Yin R., Hou D., Xue Y., Zhang M., Diao X., Zhang Y., Wu J., Hu J., Hu X. (2018). The glucose-lowering effect of Foxtail millet in subjects with impaired glucose tolerance: A self-controlled clinical trial. Nutrients.

[45] Howard B.V., Aragaki A.K., Tinker L.F., Allison M., Hingle M.D., Johnson K.C., Manson J.E., Shadyab A.H., Shikany J.M., Snetselaar L.G. (2017). A low-fat dietary pattern and diabetes: A secondary analysis from the Women’s Health Initiative Dietary Modification Trial. Diabetes Care.

[46] Marventano S., Vetrani C., Vitale M., Godos J., Riccardi G., Grosso G. (2017). Whole grain intake and glycaemic control in healthy subjects: A systematic review and meta-analysis of randomized controlled trials. Nutrients.

[47] Mofidi A., Ferraro Z.M., Stewart K.A., Tulk H.M.F., Robinson L.E., Duncan A.M., Graham T.E. (2012). The acute impact of ingestion of sourdough and whole-grain breads on blood glucose, insulin, and incretins in overweight and obese men. J. Nutr. Met..

[48] Soong Y.Y., Quek R.Y.C., Henry C.J. (2015). Glycemic potency of muffins made with wheat, rice, corn, oat and barley flours: A comparative study between *in vivo* and *in vitro*. Eur. J. Nutr..

[49] Zafar T.A., Al-Hassawi F., Al-Khulaifi F., Al-Rayyes G., Waslien C., Huffman F.G. (2013). Organoleptic and glycemic properties of chickpea-wheat composite breads. J. Food Sci. Technol..

[50] Luhovyy B.L., Mollard R.C., Yurchenko S., Nunez M.F., Berengut S., Liu T.T., Smith C.E., Pelkman C.L., Anderson G.H. (2014). The effects of whole grain high-amylose maize flour as a source of resistant starch on blood glucose, satiety, and food intake in young men. J. Food Sci..

[51] Poquette N.M., Gu X., Lee S.-O. (2014). Grain sorghum muffin reduces glucose and insulin responses in men. Food Funct..

[52] Lappi J., Aura A.-M., Katina K., Nordlund E., Kolehmainen M., Mykkänen H., Poutanen K. (2013). Comparison of postprandial phenolic acid excretions and glucose responses after ingestion of breads with bioprocessed or native rye bran. Food Funct..

[53] Bao L., Cai X., Xu M., Li Y. (2014). Effect of oat intake on glycaemic control and insulin sensitivity: A meta-analysis of randomised controlled trials. Br. J. Nutr..

[54] He L.-X., Zhao J., Huang Y.-S., Li Y. (2016). The difference between oats and beta-glucan extract intake in the management of HbA1c, fasting glucose and insulin sensitivity: A meta-analysis of randomized controlled trials. Food Funct..

[55] Sandberg J.C., Björck I.M.E., Nilsson A.C. (2017). Effects of whole grain rye, with and without resistant starch type 2 supplementation, on glucose tolerance, gut hormones, inflammation and appetite regulation in an 11–14.5 hour perspective; a randomized controlled study in healthy subjects. Nutr. J..

[56] Sang S., Chu Y. (2017). Whole grain oats, more than just a fiber: Role of unique phytochemicals. Mol. Nutr. Food Res..

[57] Shi L., Brunius C., Lindelöf M., Shameh S.A., Wu H., Lee I., Landberg R., Moazzami A.A. (2017). Targeted metabolomics reveals differences in the extended postprandial plasma metabolome of healthy subjects after intake of whole-grain rye porridges versus refined wheat bread. Mol. Nutr. Food Res..

[58] Jenkins D.J.A., Kendall C.W.C., Vuksan V., Faulkner D., Augustin L.S.A., Mitchell S., Ireland C., Srichaikul K., Mirrahimi A., Chiavaroli L. (2014). Effect of lowering the glycemic load with Canola oil on glycemic control and cardiovascular risk factors: A randomized controlled trial. Diabetes Care.

[59] Pyo Y.-H., Lee K.-W. (2014). Preventive effect of monascus-fermented products enriched with ubiquinones on Type 2 diabetic rats induced by a high-fructose plus high-fat diet. J. Med. Food.

[60] Kang R., Kim M., Chae J., Lee S.-H., Lee J. (2014). Consumption of whole grains and legumes modulates the genetic effect of the APOA5 -1131C variant on changes in triglyceride and apolipoprotein A-V concentrations in patients with impaired fasting glucose or newly diagnosed type 2 diabetes. Trials.

[61] Asemi Z., Samimi M., Tabassi Z., Esmaillzadeh A. (2014). The effect of DASH diet on pregnancy outcomes in gestational diabetes: A randomized controlled clinical trial. Eur. J. Clin. Nutr..

[62] Pereira M.A., Jacobs D.R., Pins J.J., Raatz S.K., Gross M.D., Slavin J.L., Seaquist E.R. (2002). Effect of whole grains on insulin sensitivity in overweight hyperinsulinemic adults. Am. J. Clin. Nutr..

[63] Liu J., Zhao Y., Wu Q., John A., Jiang Y., Yang J., Liu H., Yang B. (2018). Structure characterisation of polysaccharides in vegetable “okra” and evaluation of hypoglycemic activity. Food Chem..

[64] Kwon D.Y., Hong S.M., Ahn I.S., Kim Y.S., Shin D.W., Park S. (2009). Kochujang, a Korean fermented red pepper plus soybean paste, improves glucose homeostasis in 90% pancreatectomized diabetic rats. Nutrition.

[65] Ayoub H.M., McDonald M.R., Sullivan J.A., Tsao R., Platt M., Simpson J., Meckling K.A. (2017). The effect of anthocyanin-rich purple vegetable diets on metabolic syndrome in obese Zucker rats. J. Med. Food.

[66] Wu T., Luo J., Xu B. (2015). In vitro antidiabetic effects of selected fruits and vegetables against glycosidase and aldose reductase. Food Sci. Nutr..

[67] Rioux L.E., Turgeon S.L., Beaulieu M. (2007). Characterization of polysaccharides extracted from brown seaweeds. Carbohydr. Polym..

[68] Hu J.-L., Nie S.-P., Xie M.-Y. (2018). Antidiabetic mechanism of dietary polysaccharides based on their gastrointestinal functions. J. Agric. Food Chem..

[69] Kumar G., Sharmila Banu G., Ganesan Murugesan A., Pandian M.R. (2007). Antihyperglycaemic and antiperoxidative effect of *Helicteres isora* L. bark extracts in streptozotocin-induced diabetic rats. J. Appl. Biomed..

[70] Kumar G., Sharmila Banu G., Ganesan Murugesan A. (2008). Effect of *Helicteres isora* bark extracts on heart antioxidant status and lipid peroxidation in streptozotocin diabetic rats. J. Appl. Biomed..

[71] Wang Q., Wu X., Shi F., Liu Y. (2019). Comparison of antidiabetic effects of saponins and polysaccharides from *Momordica charantia* L. in STZ-induced type 2 diabetic mice. Biomed. Pharmacother..

[72] Kumar Bhateja P., Singh R. (2014). Antidiabetic activity of *Acacia tortilis* (Forsk.) Hayne ssp. raddiana polysaccharide on streptozotocin-nicotinamide induced diabetic rats. BioMed Res. Int..

[73] Wang L., Wu H., Chang N., Zhang K. (2010). Anti-hyperglycemic effect of the polysaccharide fraction isolated from *Mactra veneriformis*. Front Chem. Sci. Eng..

[74] Zhao R., Qiu B., Li Q., Zhang T., Zhao H., Chen Z., Cai Y., Ruan H., Ge W., Zheng X. (2014). LBP-4a improves insulin resistance via translocation and activation of GLUT4 in OLETF rats. Food Funct..

[75] Jin H., Zhang Y.-J., Jiang J.-X., Zhu L.-Y., Chen P., Li J., Yao H.-Y. (2013). Studies on the extraction of pumpkin components and their biological effects on blood glucose of diabetic mice. J. Food Drug Anal..

[76] Jiao Y., Wang X., Jiang X., Kong F., Wang S., Yan C. (2017). Antidiabetic effects of *Morus alba* fruit polysaccharides on high-fat diet- and streptozotocin-induced type 2 diabetes in rats. J. Ethnopharmacol..

[77] Lei H., Guo S., Han J., Wang Q., Zhang X., Wu W. (2012). Hypoglycemic and hypolipidemic activities of MT-α-glucan and its effect on immune function of diabetic mice. Carbohydr. Polym..

[78] Huang H.-Y. (2014). Effect of *Pleurotus tuber-regium* polysaccharides supplementation on the progression of diabetes complications in obese-diabetic rats. Chin. J. Physiol..

[79] Leslie W.S., Taylor R., Harris L., Lean M.E.J. (2016). Weight losses with low-energy formula diets in obese patients with and without type 2 diabetes: Systematic review and meta-analysis. Int. J. Obesity..

[80] Anoop S., Misra A., Bhatt S.P., Gulati S., Mahajan H. (2019). High fasting C-peptide levels and insulin resistance in non-lean & non-obese (BMI > 19 to < 25 kg/m^2^) Asian Indians with type 2 diabetes are independently associated with high intra-abdominal fat and liver span. Diabet. Met. Synd. Clin. Res. Rev..

[81] Ganesan K., Xu B. (2018). Anti-obesity effects of medicinal and edible mushrooms. Molecules.

[82] Vasu S., McClenaghan N.H., McCluskey J.T., Flatt P.R. (2014). Mechanisms of toxicity by proinflammatory cytokines in a novel human pancreatic beta-cell line, 1.1B4. Biochim. Biophy. Acta.

[83] Franz M.J., Boucher J.L., Rutten-Ramos S., VanWormer J.J. (2015). Lifestyle weight-loss intervention outcomes in overweight and obese adults with type 2 diabetes: A systematic review and meta-analysis of randomized clinical trials. J. Acad. Nutr. Diet..

[84] Zhang Y., Ren C., Lu G., Mu Z., Cui W., Gao H., Wang Y. (2014). Anti-diabetic effect of mulberry leaf polysaccharide by inhibiting pancreatic islet cell apoptosis and ameliorating insulin secretory capacity in diabetic rats. Int. Immunopharmacol..

[85] Zhu K.-X., Nie S.-P., Li C., Gong D., Xie M.-Y. (2014). *Ganoderma atrum* polysaccharide improves aortic relaxation in diabetic rats via PI3K/Akt pathway. Carbohydr. Polym..

[86] Zhu K., Nie S., Li C., Lin S., Xing M., Li W., Gong D., Xie M. (2013). A newly identified polysaccharide from *Ganoderma atrum* attenuates hyperglycemia and hyperlipidemia. Int. J. Biol. Macromol..

[87] Zheng J., Yang B., Yu Y., Chen Q., Huang T., Li D. (2012). *Ganoderma lucidum* polysaccharides exert anti-hyperglycemic effect on streptozotocin-induced diabetic rats through affecting & beta-cells. Comb. Chem. High Throughput Screen..

[88] Li F., Zhang Y., Zhong Z. (2011). Antihyperglycemic Effect of *Ganoderma lucidum* polysaccharides on streptozotocin-induced diabetic mice. Int. J. Mol. Sci..

[89] Zhu H.-Y., Chen G.-T., Meng G.-L., Xu J.-L. (2015). Characterization of pumpkin polysaccharides and protective effects on streptozotocin-damaged islet cells. Chin. J. Natl. Med..

[90] Hu S., Wang J., Xu H., Wang Y., Li Z., Xue C. (2014). Fucosylated chondroitin sulphate from sea cucumber inhibits high-fat-sucrose diet-induced apoptosis in mouse pancreatic islets via down-regulating mitochondrial signaling pathway. J. Funct. Foods.

[91] Kumar G., Banu G.S., Murugesan A.G., Pandian M.R. (2006). Hypoglycaemic effect of *Helicteres isora* bark extract in rats. J. Ethnopharmacol..

[92] Kumar G., Sharmila Banu G., Murugesan A.G., Rajasekara Pandian M. (2007). Effect of *Helicteres isora*. Bark extracts on brain antioxidant status and lipid peroxidation in streptozotocin diabetic rats. Pharm. Biol..

[93] Islam T., Ganesan K., Xu B. (2019). New Insight into mycochemical profiles and antioxidant potential of edible and medicinal mushrooms: A review. Int. J. Med. Mushrooms.

[94] Kumar G., Maheswaran R., Sharmila Banu G. (2013). Antihyperlipideamic effect of *Solanum trilobatum* L. leaves extract on streptozotocin induced diabetic rats. Asian J. Biomed. Pharma. Sci..

[95] Zhang T., Jayachandran M., Ganesan K., Xu B. (2018). Black Truffle aqueous extract attenuates oxidative stress and inflammation in STZ-induced hyperglycemic rats via Nrf2 and NF-κB pathways. Front. Pharmacol..

[96] Jiang Y., Wang L., Zhang L., Wang T., Zhou Y., Ding C., Yang R., Wang X., Yu L. (2015). Optimization of extraction and antioxidant activity of polysaccharides from *Salvia miltiorrhiza* Bunge residue. Int. J. Biol. Macromol..

[97] Kou L., Du M., Liu P., Zhang B., Zhang Y., Yang P., Shang M., Wang X. (2018). Anti-diabetic and anti-nephritic activities of *Grifola frondosa* mycelium polysaccharides in diet-streptozotocin-induced diabetic rats via modulation on oxidative stress. Appl. Biochem. Biotechnol..

[98] Chen X., Tang J., Xie W., Wang J., Jin J., Ren J., Jin L., Lu J. (2013). Protective effect of the polysaccharide from *Ophiopogon japonicus* on streptozotocin-induced diabetic rats. Carbohydr. Polym..

[99] Liu Y., Wan L., Xiao Z., Wang J., Wang Y., Chen J. (2013). Antidiabetic activity of polysaccharides from tuberous root of *Liriope spicata* var. *prolifera* in KKAy Mice. Evid. Based Complement. Alternat. Med..

[100] Chen C., Huang Q., Li C., Fu X. (2017). Hypoglycemic effects of a *Fructus mori* polysaccharide *in vitro* and *in vivo*. Food Funct..

[101] Sun C., Chen Y., Li X., Tai G., Fan Y., Zhou Y. (2014). Anti-hyperglycemic and anti-oxidative activities of ginseng polysaccharides in STZ-induced diabetic mice. Food Funct..

[102] Ganeshpurkar A., Kohli S., Rai G. (2014). Antidiabetic potential of polysaccharides from the white oyster culinary-medicinal mushroom *Pleurotus florida* (Higher Basidiomycetes). Int. J. Med. Mushrooms.

[103] Yang Z.W., Ouyang K.H., Zhao J., Chen H., Xiong L., Wang W.J. (2016). Structural characterization and hypolipidemic effect of *Cyclocarya paliurus* polysaccharide in rat. Int. J. Biol. Macromol..

[104] Wu Y., Zhou F., Jiang H., Wang Z., Hua C., Zhang Y. (2018). Chicory (*Cichorium intybus* L.) polysaccharides attenuate high-fat diet-induced non-alcoholic fatty liver disease via AMPK activation. Int. J. Biol. Macromol..

[105] Jia L., Li W., Li J., Li Y., Song H., Luan Y., Qi H., Ma L., Lu X., Yang Y. (2016). *Lycium barbarum* polysaccharide attenuates high-fat diet-induced hepatic steatosis by up-regulating SIRT1 expression and deacetylase activity. Sci. Rep..

[106] Ren R., Gong J., Zhao Y., Zhuang X., Ye Y., Huang F., Lin W. (2018). Sulfated polysaccharide from *Enteromorpha prolifera* suppresses SREBP-1c and ACC expression to lower serum triglycerides in high-fat-diet-induced hyperlipidaemic rats. J. Funct. Foods.

[107] Nie Y., Luo F., Wang L., Yang T., Shi L., Li X., Shen J., Xu W., Guo T., Lin Q. (2017). Anti-hyperlipidemic effect of rice bran polysaccharide and its potential mechanism in high-fat diet mice. Food Funct..

[108] Bruce C.R., Hoy A.J., Turner N., Watt M.J., Allen T.L., Carpenter K., Cooney G.J., Febbraio M.A., Kraegen E.W. (2009). Overexpression of carnitine palmitoyltransferase-1 in skeletal muscle is sufficient to enhance fatty acid oxidation and improve high-fat diet–induced insulin resistance. Diabetes.

[109] Li Y., Yuan Y., Lei L., Li F., Zhang Y., Chen J., Zhao G., Wu S., Yin R., Ming J. (2017). Carboxymethylation of polysaccharide from *Morchella angusticepes* Peck enhances its cholesterol-lowering activity in rats. Carbohydr. Polym..

[110] Gil-Ramírez A., Caz V., Smiderle F.R., Martin-Hernandez R., Largo C., Tabernero M., Marín F.R., Iacomini M., Reglero G., Soler-Rivas C. (2016). Water-soluble compounds from *Lentinula edodes* influencing the HMG-CoA reductase activity and the expression of genes involved in the cholesterol metabolism. J. Agric. Food Chem..

[111] Park J., Yeom M., Hahm D.H. (2016). Fucoidan improves serum lipid levels and atherosclerosis through hepatic SREBP-2-mediated regulation. J. Pharmacol. Sci..

[112] Musunuru K., Kathiresan S. (2016). Surprises from genetic analyses of lipid risk factors for atherosclerosis. Circ. Res..

[113] Lewington S., Whitlock G., Clarke R., Sherliker P., Emberson J., Halsey J., Qizilbash N., Peto R., Collins R., Prospective Studies Collaboration (2007). Blood cholesterol and vascular mortality by age, sex, and blood pressure: A meta-analysis of individual data from 61 prospective studies with 55,000 vascular deaths. Lancet.

[114] Petroglou D., Kanellos I., Savopoulos C., Kaiafa G., Chrysochoou A., Skantzis P., Daios S., Hatzitolios A.I., Giannoglou G. (2018). The LDL-receptor and its molecular properties: From theory to novel biochemical and pharmacological approaches in reducing LDL-cholesterol. Curr. Med. Chem..

[115] Farnier M. (2013). PCSK9 inhibitors. Curr. Opin. Lipidol..

[116] Li W., Li Y., Wang Q., Yang Y. (2014). Crude extracts from *Lycium barbarum* suppress SREBP-1c expression and prevent diet-induced fatty liver through AMPK activation. BioMed Res. Int..

[117] Yang M., Li X., Zeng X., Ou Z., Xue M., Gao D., Liu S., Li X., Yang S. (2016). *Rheum palmatum* L. Attenuates High Fat Diet-Induced Hepatosteatosis by Activating AMP-Activated Protein Kinase. Am. J. Chin. Med..

[118] Wang C.M., Yuan R.S., Zhuang W.Y., Sun J.H., Wu J.Y., Li H., Chen J.G. (2016). Schisandra polysaccharide inhibits hepatic lipid accumulation by down-regulating the expression of SREBPs in NAFLD mice. Lipids Health Dis..

[119] Huang X., Tang J., Zhou Q., Lu H., Wu Y., Wu W. (2010). Polysaccharide from fuzi (FPS) prevents hypercholesterolemia in rats. Lipids Health Dis..

[120] Kim H., Wang Q., Shoemaker C.F., Zhong F., Bartley G.E., Yokoyama W.H. (2014). Polysaccharide gel coating of the leaves of *Brasenia schreberi* lowers plasma cholesterol in hamsters. J. Tradit. Complement. Med..

[121] Jayachandran M., Xiao J., Xu B. (2017). A critical review on health promoting benefits of edible mushrooms through gut microbiota. Int. J. Mol. Sci..

[122] Westwell-Roper C., Dai D.L., Soukhatcheva G., Potter K.J., van Rooijen N., Ehses J.A., Verchere C.B. (2011). IL-1 Blockade attenuates islet amyloid polypeptide-induced proinflammatory cytokine release and pancreatic islet graft dysfunction. J. Immunol..

[123] Wang K., Cao P., Shui W., Yang Q., Tang Z., Zhang Y. (2015). *Angelica sinensis* polysaccharide regulates glucose and lipid metabolism disorder in prediabetic and streptozotocin-induced diabetic mice through the elevation of glycogen levels and reduction of inflammatory factors. Food Funct..

[124] Zhou J., Xu G., Yan J., Li K., Bai Z., Cheng W., Huang K. (2015). *Rehmannia glutinosa* (Gaertn.) DC. polysaccharide ameliorates hyperglycemia, hyperlipemia and vascular inflammation in streptozotocin-induced diabetic mice. J. Ethnopharmacol..

[125] Zhou J., Yan J., Bai Z., Li K., Huang K. (2015). Hypoglycemic activity and potential mechanism of a polysaccharide from the loach in streptozotocin-induced diabetic mice. Carbohydr. Polym..

[126] Hu J., Pang W., Chen J., Bai S., Zheng Z., Wu X. (2013). Hypoglycemic effect of polysaccharides with different molecular weight of *Pseudostellaria heterophylla*. BMC Complement. Altern. Med..

[127] Kanagasabapathy G., Kuppusamy U.R., Abd Malek S.N., Abdulla M.A., Chua K.-H., Sabaratnam V. (2012). Glucan-rich polysaccharides from *Pleurotus sajor-caju* (Fr.) Singer prevents glucose intolerance, insulin resistance and inflammation in C57BL/6J mice fed a high-fat diet. BMC Complement. Altern. Med..

[128] Guo C., Li R., Zheng N., Xu L., Liang T., He Q. (2013). Anti-diabetic effect of ramulus mori polysaccharides, isolated from *Morus alba* L. on STZ-diabetic mice through blocking inflammatory response and attenuating oxidative stress. Int. Immunopharmacol..

[129] Yu W., Chen H., Xiang Z., He N. (2019). Preparation of polysaccharides from *Ramulus mori*, and their antioxidant, anti-inflammatory and antibacterial activities. Molecules.

[130] Zhang J.-G., Liu Q., Liu Z.-L., Li L., Yi L.-T. (2015). Antihyperglycemic activity of *Anoectochilus roxburghii* polysaccharose in diabetic mice induced by high-fat diet and streptozotocin. J. Ethnopharmacol..

[131] Hou D., Yousaf L., Xue Y., Hu J., Wu J., Hu X., Feng N., Shen Q. (2019). Mung bean (*Vigna radiata* L.): Bioactive polyphenols, polysaccharides, peptides, and health benefits. Nutrients.

[132] Cheng F., Yan X., Zhang M., Chang M., Yun S., Meng J., Liu J., Feng C.-P. (2017). Regulation of RAW 264.7 cell-mediated immunity by polysaccharides from *Agaricus blazei* Murill via the MAPK signal transduction pathway. Food Funct..

[133] Wang S., Li Q., Zang Y., Zhao Y., Liu N., Wang Y., Xu X., Liu L., Mei Q. (2017). Apple polysaccharide inhibits microbial dysbiosis and chronic inflammation and modulates gut permeability in HFD-fed rats. Int. J. Biol. Macromol..

[134] Zhang H., Zhong J., Zhang Q., Qing D., Yan C. (2019). Structural elucidation and bioactivities of a novel arabinogalactan from *Coreopsis tinctoria*. Carbohydr. Polym..

[135] Xu Y., Niu X., Liu N., Gao Y., Wang L., Xu G., Li X., Yang Y. (2018). Characterization, antioxidant and hypoglycemic activities of degraded polysaccharides from blackcurrant (*Ribes nigrum* L.) fruits. Food Chem..

[136] Meng Q., Chen F., Xiao T., Zhang L. (2019). Inhibitory effects of polysaccharide from *Diaphragma juglandis fructus* on α-amylase and α-d-glucosidase activity, streptozotocin-induced hyperglycemia model, advanced glycation end-products formation, and H_2_O_2_-induced oxidative damage. Int. J. Biol. Macromol..

[137] Senthil S., Chandrasekaran R., Arjun H.A., Anantharaman P. (2019). *In vitro* and *in silico* inhibition properties of fucoidan against α-amylase and α-d-glucosidase with relevance to type 2 diabetes mellitus. Carbohydr. Polym..

[138] Cui J., Gu X., Wang F., Ouyang J., Wang J. (2015). Purification and structural characterization of an α-glucosidase inhibitory polysaccharide from apricot (*Armeniaca sibirica* L. Lam.) pulp. Carbohydr. Polym..

[139] Hu J.-L., Nie S.-P., Li C., Xie M.-Y. (2013). *In vitro* effects of a novel polysaccharide from the seeds of *Plantago asiatica* L. on intestinal function. Int. J. Biol. Macromol..

[140] Lakshmanasenthil S., Vinothkumar T., Geetharamani D., Marudhupandi T., Suja G., Sindhu N.S. (2014). Fucoidan—A novel α-amylase inhibitor from *Turbinaria ornata* with relevance to NIDDM therapy. Biocatal. Agric. Biotechnol..

[141] Vinoth Kumar T., Lakshmanasenthil S., Geetharamani D., Marudhupandi T., Suja G., Suganya P. (2015). Fucoidan—A α-d-glucosidase inhibitor from *Sargassum wightii* with relevance to type 2 diabetes mellitus therapy. Int. J. Biol. Macromol..

[142] Moullé V.S., Ghislain J., Poitout V. (2017). Nutrient regulation of pancreatic β-cell proliferation. Biochimie.

[143] Liu Y., Li X., Xie C., Luo X., Bao Y., Wu B., Hu Y., Zhong Z., Liu C., Li M. (2016). Prevention effects and possible molecular mechanism of mulberry leaf extract and its formulation on rats with insulin-insensitivity. PLoS ONE.

[144] Wang L.-Y., Wang Y., Xu D.-S., Ruan K.-F., Feng Y., Wang S. (2012). MDG-1, a polysaccharide from *Ophiopogon japonicus* exerts hypoglycemic effects through the PI3K/Akt pathway in a diabetic KKAy mouse model. J. Ethnopharmacol..

[145] Xiao Z.-Q., Wang Y.-L., Gan S.-R., Chen J.-C. (2013). Polysaccharides from *Liriopes radix* ameliorates hyperglycemia via various potential mechanisms in diabetic rats. J. Sci. Food Agric..

[146] Zhu K.-X., Nie S.-P., Tan L.-H., Li C., Gong D.-M., Xie M.-Y. (2016). A polysaccharide from *Ganoderma atrum* Improves liver function in type 2 diabetic rats via antioxidant action and short-chain fatty acids excretion. J. Agric. Food Chem..

[147] Lin W., Wang W., Liao D., Chen D., Zhu P., Cai G., Kiyoshi A. (2015). A polysaccharides from *Enteromorpha prolifera* improve glucose metabolism in diabetic rats. J. Diabet. Res..

[148] Ma X., Zhou F., Chen Y., Zhang Y., Hou L., Cao X., Wang C. (2014). A polysaccharide from *Grifola frondosa* relieves insulin resistance of HepG2 cell by Akt-GSK-3 pathway. Glycoconj. J..

[149] Zhao C., Liao Z., Wu X., Liu Y., Liu X., Lin Z., Huang Y., Liu B. (2014). Isolation, purification, and structural features of a polysaccharide from *Phellinus linteus* and Its hypoglycemic effect in alloxan-induced diabetic mice. J. Food Sci..

[150] Hu S., Chang Y., Wang J., Xue C., Li Z., Wang Y. (2013). Fucosylated chondroitin sulfate from sea cucumber in combination with rosiglitazone improved glucose metabolism in the liver of the insulin-resistant mice. Biosci. Biotechnol. Biochem..

[151] Hu S., Xu H., Chen R., Wang J., Li Z., Xu J. (2014). Activation of PKB and ERK, but not PI3K, is involved in fucosylated chondroitin sulphate from *Acaudina molpadioides* induced glucose uptake. J. Funct. Foods.

[152] Liao Z., Zhang J., Liu B., Yan T., Xu F., Xiao F., Wu B., Bi K., Jia Y. (2019). Polysaccharide from okra (*Abelmoschus esculentus* (L.) Moench) improves antioxidant capacity via PI3K/AKT Pathways and Nrf2 translocation in a type 2 diabetes model. Molecules.

[153] Ye H., Shen Z., Cui J., Zhu Y., Li Y., Chi Y., Wang J., Wang P. (2019). Hypoglycemic activity and mechanism of the sulfated rhamnose polysaccharides chromium(III) complex in type 2 diabetic mice. Bioorg. Chem..

[154] Chen Y., Liu D., Wang D., Lai S., Zhong R., Liu Y., Yang C., Liu B., Sarker M.R., Zhao C. (2019). Hypoglycemic activity and gut microbiota regulation of a novel polysaccharide from *Grifola frondosa* in type 2 diabetic mice. Food Chem. Toxicol..

[155] Xu L., Yang F., Wang J., Huang H., Huang Y. (2015). Anti-diabetic effect mediated by *Ramulus mori* polysaccharides. Carbohydr. Polym..

